# The circadian clock gene CYCLE as a potential target for disrupting blood-feeding behavior in the mosquito *Culex pipiens*

**DOI:** 10.1371/journal.pntd.0014218

**Published:** 2026-04-21

**Authors:** Chanuka Wijewardana, Yunxuan Chen, Chenxuan Jiang, Yinghui Zhou, Yuhan Guo, Donghui Zhang, Lu Chen, Min Hou, Zhipeng Xu, Yaping Hu, MinJun Ji, Lin Chen

**Affiliations:** 1 Department of Pathogen Biology, School of Basic Medical Sciences, Nanjing Medical University, Nanjing, Jiangsu, China; 2 Department of Clinical Laboratory, Sir Run Run Hospital, Nanjing Medical University, Nanjing, Jiangsu, China; 3 Department of Teaching Management, Nanjing Medical University, Nanjing, Jiangsu, China; 4 Key Laboratory for Infections and Control of Jiangsu Province, Nanjing Medical University, Nanjing, Jiangsu, China; 5 Scientific Observation and Research Station for Ecological Environment of Wuyi Mountains, Ministry of Ecology and Environment, China/Fujian Wuyishan State Integrated Monitoring Station for Ecological Quality of Forest Ecosystem/Nanjing Institute of Environmental Sciences, Ministry of Ecology and Environment, Nanjing, Jiangsu, China; University of Cincinnati, UNITED STATES OF AMERICA

## Abstract

**Background:**

*C. pipiens* blood feeding rhythm exhibits a circadian rhythm that have the ability to persist under absent of environmental cues, consistent with endogenous control. Variations observed in the blood feeding rhythm is accompanied by changes in CYCLE (CYC) expression and olfactory sensitivity, supporting a role for CYC in pathways related to feeding propensity control. In our study, we explore the endogenous circadian control of blood feeding and the effects of mistimed feeding.

**Results:**

The blood-feeding rhythm of *C. pipiens* is governed by an endogenous circadian clock, wherein the variations observed in blood feeding propensity is accompanied by coordinated changes in CYCLE expression and the olfactory sensitivity. We observed a light-induced masking effect that can elevate feeding propensity in subjective daytime in the absence of light, suggesting acute involvement of light in feeding regulation. Under extended starvation induced molecular stress, olfactory sensitivity was reduced along with variations in CYC expression, that is consistent with an olfactory shutdown under extreme metabolic stress. The proposed mechanism placed CYC as a molecular mediator to shut down the olfactory system in mosquitoes in response to extreme metabolic stress. We confirmed through CYCLE knockdown and behavioral assays that the loss of function of CYCLE can alter the feeding propensity in mosquitoes. We also found that mistimed feeding severely compromises mosquito reproductive health and notably evokes compensatory mechanisms.

**Conclusions:**

This study provides evidence for endogenous regulation in the blood-feeding behavior of *C. pipiens*, from host-seeking through olfaction to reproductive fitness. The observed changes in blood feeding rhythm are associate with the light conditions provided, and the underlying rhythm is governed through an internal clock, including core clock component CYCLE and accompanied by the rhythmic changes in small neuropeptide F expression. Through our study, we provide insights into complex genetics involved in chronobiology that control mosquito biting behaviour and reveal a novel target in chronobiology-based vector control strategies aimed at reducing mosquito-human contact.

## Author summary

Mosquitoes are key vectors for diseases like West Nile virus, and understanding how they time their feeding behavior can reveal control points for intervention. In this study, we explored the feeding rhythm of *Culex pipiens*, a major vector mosquito species. We found that this mosquito has a strong nocturnal feeding pattern regulated by its internal clock, which can be influenced by light, through a process known as “masking.” Light exposure suppressed feeding even when mosquitoes would normally be active, revealing the flexibility of their behavior in response to environmental cues. We identified the core clock gene CYC as a key regulator of feeding propensity. Knockdown of CYC altered the feeding propensities at day and night timepoint anchors used but did not abolish the nocturnal feeding window, suggesting presence of other compensatory mechanisms. Additionally, we showed that feeding at inappropriate times disrupts digestion and reduces egg production, highlighting the importance of timing for mosquito fitness. Our findings contribute to understanding how environmental and internal cues control mosquito behavior.

## Introduction

*Culex pipiens* is a medically important member of the Culicidae family that exhibits several circadian behaviors influencing key moments in the life cycle, such as mating and flight. *Culex pipiens pallens* is anautogenous, requiring a vertebrate blood meal to initiate vitellogenesis, whereas the autogenous *Culex pipiens molestus* does not. This fundamental difference underscores the ecological and physiological importance of blood-feeding rhythms [1,2]. Most of these rhythms are nocturnal, synchronized by environmental cues such as light, temperature, and humidity [3–5]. In molecular basis, these rhythms are controlled through a central transcription-translation feedback loop (TTFL) that is consist of core clock genes such as CLOCK (CLK), CYCLE (CYC), PERIOD (PER), and TIMELESS (TIM), and a blue light photoreceptor such as Cryptochrome 2 (CRY2) that mediate gene expression depending on the external light conditions received [6–8]. Comparative genomic studies reveal high conservation of circadian genes between *Drosophila* and mosquitoes, validating the use of these model organisms as a starting point for investigating vector chronobiology [9–15].

Circadian rhythms are essential to synchronize mosquito behaviors with optimal environmental conditions. For example, blood-feeding in *Culex spp.* peaks during early scotophase, this peak aligns with the host inactivity and minimized risk from predators, ensuring a successful blood meal acquisition and survivability [4,16–18]. These behaviors are affected and controlled by internal conditions and external zeitgebers, such as nutritional status, insemination, and sometimes parasites like filarial worms and *Plasmodium*, which show signs of co-evolution to match the vector’s activity patterns for successful transmission [7,19,20]. In addition, core clock genes like CYC and CRY2 have been linked to behaviors like mating and diapause, which suggest that a well-regulated clock is vital for mosquito fitness and survival in changing environmental conditions [6,21]. Rapid urbanization has caused the emergence of hybrid strains between *C. pallens* and *C. molestus*, leading to broader host preferences that diverge from the host preferences unique to each strain and complicating the understanding of behavioral patterns and vector control efforts that incorporate rhythms [22,23]. Despite having different host preferences (*C. pallens* preferring avian hosts and *C. molestus* preferring mammalian hosts), both strains maintain similar feeding rhythms, implying that rhythm is mainly driven by host availability rather than host preference [4,24,25]. While the blood feeding rhythm in mosquitoes is known to follow a rhythmic pattern in accordance with time of the day and lighting conditions, it is unknown how the circadian clock genes are integrated to maintain this rhythmic pattern using external environmental cues and the metabolic state of mosquitoes as references.

Host-seeking behavior is largely governed by olfactory cues, such as 1-octen-3-ol, which enable mosquitoes to differentiate between and locate preferred hosts. CO_2_ also plays a role in long-range host seeking [26–28]. The olfactory system is under circadian control, with peak gene expression during active periods to conserve energy [29,30]. Behavioral plasticity allows mosquitoes to adapt to changes in host availability through olfactory learning. This enables them to adjust odorant receptor sensitivity and shift host preference following repeated unsuccessful encounters [31]. Feeding rhythms also show flexibility under metabolic stresses such as starvation, indicating a mechanism that bypasses the core clock controls as a response to the adverse conditions to ensure survival [4]. Understandings on different mosquito systems lead to a dual-system hypothesis, which links locomotor timing and olfactory sensitivity, ultimately suggesting that feeding rhythms are co-regulated, a theory with implications for disrupting mosquito-host interactions in vector control [21,31].

Locomotion rhythms define the window for feeding activities, particularly in females seeking blood meals after insemination [32]. Activity patterns are orchestrated by circadian mechanisms and modulated by factors such as mating, temperature, and light conditions [33,34]. In *Aedes aegypti*, the lights-off cue triggers the flight and host-seeking behavior, supporting the idea that scotophase transitions serve as activators for feeding-related locomotion [35,36]. The interplay between internal clocks and environmental disruptions, such as artificial light at night, can alter feeding timing and fluctuate feeding propensity, raising concerns about disease transmission in rapidly urbanized settings [37,38]. Therefore, understanding how circadian pathways integrate sensory and locomotor cues offers novel opportunities for targeted mosquito control strategies, especially as studies confirm that the majority of clock-regulated behaviors are conserved across vector species like *Culex quinquefasciatus* and *Aedes albopictus* [6].

This study aims at dissecting the complex mechanisms involved in chronobiology that govern the blood-feeding behavior in mosquitoes, identifying the core clock components that regulate the blood-feeding propensity by integrating environmental cues and internal environment conditions of mosquitoes, such as metabolic stress, and evaluating the physiological consequences of mistimed feeding on mosquitoes to identify a potential weak point in chronobiology to exploit in vector control strategies.

## Materials and methods

### Mosquito rearing

Laboratory-maintained *C. pipiens pallens* strains were kept under standard conditions of 27 ± 1°C temperature and at 70% humidity in an incubator isolated from the external environment. The mosquitoes were blood-fed at 1-week intervals with restrained mice to obtain the eggs to maintain the generation. Mosquito larvae were held in dechlorinated water and provided with pulverized rat food. All of the larvae and pupae were held in an isolated environment under standard 12- hour light and dark cycles and at the same temperature of 27 ± 1°C and at 70% humidity. Adult mosquitoes were provided with a 10% glucose diet and held in cages of 10 cm x 10 cm x 30 cm under the same standard light and dark cycles as larvae and pupae.

### Light training

Light training of 12 hrs. light: 12 hrs. dark conditions (LDLD) and reversed light conditions (DLDL) were carried out as mentioned by Liu *et al* [6], with notable differences where light training time was reduced from four days to three days. During the light training, the mosquitoes were provided with a 10% glucose solution. Second day post eclosion LD group continued the standard LD cycle (12h. light and dark) while DL group started reverse light training with reversed 12 hrs. dark and light cycle. For the duration of three days of light training, the mosquitoes were kept as a colony to recreate their natural environment as best as possible, and on the fourth day, five days post eclosion, the females were separated and continued on separate light training as required by the experiments.

### Feeding assays

**Fig 1:**
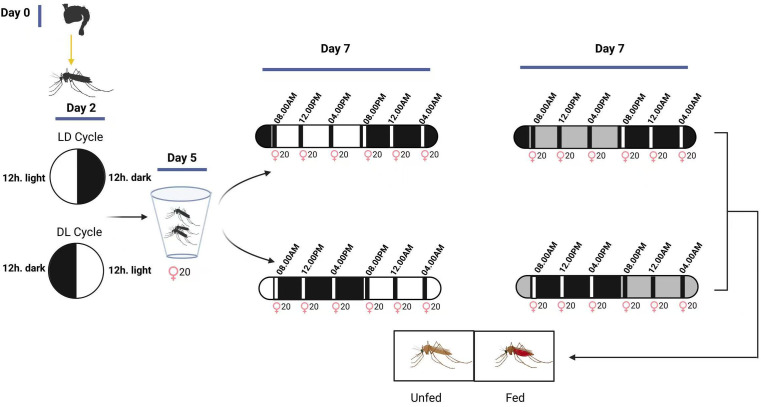
Experimental design of feeding assay. On the 2^nd^ day PE mosquitoes were grouped into each of the light training cycles, LD and DL and continued for three days then an additional day in the same or continuous darkness depending on groups LDLD or LDDD before starting the feeding assay **LD** - Typical 12 hrs. light and dark cycle, **DL** - Reversed light dark cycle, **White** - Light on period, **Black** - Light off period, **Grey** - Light off period under subjective light period. The corresponding zeitgeber timepoints are; 08.00AM - ZT0, 12.00PM - ZT4, 04.00PM - ZT8, 08.00PM - ZT12, 12.00AM - ZT16, 04.00AM - ZT20. Fig 1 was drawn using Biorender (https://www.biorender.com).

Feeding assays were utilized under different light conditions to determine the feeding rhythms. Groups of one week old (seven days post eclosion) female mosquitoes (~ 20) that were kept on 10% glucose were provided access to a restrained mouse for 15–20 minutes at 08.00AM (ZT0), 12.00PM (ZT4), 04.00PM (ZT8), 08.00PM (ZT12), 12.00AM (ZT16), and 04.00AM (ZT20), and assessed the fed conditions visually. Presence of an engorged abdomen with visible blood inside is used as the visible reference for the fed condition. Feeding rate was defined as the proportion of the mosquitoes that were visually assessed to be fallen under fed condition. LD trained mosquitoes went under the feeding assay under standard conditions of LDLD or in constant darkness following LD training (LDDD) and DL trained mosquitoes were assayed under the reversed light-dark cycle (DLDL) or in constant darkness following DL training (DLDD). (Fig 1) RNA interference group mosquitoes at 3 days post-injection (for maximum efficiency), underwent 2 days of light training. Feeding propensity for this experiment was measured at ZT4, ZT8, ZT16 and ZT20, the non-transition sampling points used for rhythm comparisons. To ensure maximum feeding, males and females were held together and starved for 4 hours before the feeding assay.

### RNA extraction and preparation for quantitative PCR

Mosquito heads were selected as the tissue site. Female mosquitoes that were under light training were extracted at different time points of the day under both LDLD and LDDD conditions: ZT0, ZT4, ZT8, ZT12, ZT16, and ZT20. Once extracted, the samples were quickly frozen at -20°C for 3 minutes to halt the transcription process and then decapitated in phosphate-buffered saline (PBS) (LABGIC Co Ltd, Beijing. CAT no. BL601A). All sampling for RNA extraction was carried out in three biological replicates for each time point. They were immediately transferred to 300 μL of ice-cold Trizol reagent (RNAex pro, Accurate biology co. Ltd, CAT no. AG21101). 10 mosquito heads were collected per tube. The samples were then held at -80°C till RNA extraction. RNA extraction was performed using the phenol: chloroform method according to the standard procedure. For quantitative PCR, cDNA synthesis was carried out using Prime Script (Takara Bio co. Ltd, CAT no. RR037A) reverse transcriptase following the standard protocol. Once the cDNA was synthesized, the final concentration was adjusted to 100ng/μl and then utilized in the qPCR. Tissue sites for Trypsin expressions and vitellogenin expressions were the midgut and the fat body, respectively.

### Quantitative PCR

The quantitative PCR was carried out using the SYBR Green Pro Taq HS Premix (Accurate Biology co. Ltd, CAT no. AG11701) following the manufacturer’s instructions. The Primers for the genes that were tested were obtained through previous work and through the NCBI primer blast program. Some primers were originally designed for *Culex quinquefasciatus,* but were decided to use due to the close genetic relation between *C. pipiens*. The 384-well PCR plates were run in a 384-plate light cycler (Quantstudio 5, Applied Biosystems co. Ltd, CAT no. A28569) under the manufacturer’s program. RPS6 was used as the internal housekeeping gene, which was tested for its stable expression in *C. pipiens*. Primers utilized in the study are presented in S1 Table. All the primers were synthesized by Genscript Biotech co. Ltd, Nanjing, China.

### Three-cage olfactory assay

A three-cage olfactory assay was utilized to measure the olfactory response of mosquitoes at different times of the day. Around 40 female mosquitoes were utilized per run, that were kept on 10% glucose diet. Mosquitoes were held in a transparent holding cage (30 cm x 30 cm x 30 cm), which was connected to two outer cages of the same dimensions, which were connected to two flasks containing olfactory sources, test (mouse) and control (distilled water). Outside air filtered through an active carbon filter was provided at a rate of 1L/min to flasks that contain the olfactory sources. After a 30-minute acclimatization window, gates to the outer cages were opened, and a 20-minute window was provided to choose a cage. Results were divided into test, control, and not chosen. For analysis, the control and non-chosen groups were combined. ZT4 served as a baseline. All conditions were standard mosquito rearing conditions.

### RNA interference

The small interfering RNA (siRNA) was used for RNA interference. The sequences were synthesized (Shanghai Genepharma co. Ltd.), and 0.5OD of the sequence was diluted in 3.3μl of DEPC water (Shanghai Genepharam co. Ltd). The diluted siRNA solution was injected into 12 hours post-emerged female mosquitoes at 69nl per mosquito in the thorax region (Nanoject III, Drummond scientific co. Ltd, CAT no. 3-000-207). A non-targeting scrambled siRNA diluted to the same concentration was used as a negative control. Once injected, the mosquitoes were transferred to paper cups and held under normal conditions. The effect of siRNA on the targeted gene was tested on the 3^rd^ day post-injection using RTqPCR (SYBR Green Pro Taq HS Premix, Accurate Biology co. Ltd, CAT no. AG11701) following RNA extraction (RNAex pro, Accurate biology co. Ltd, CAT no. AG21101) using manufacturer suggested protocols, and the sequence that showed the most efficiency was utilized in the follow-up experiments. The sequences utilized in the study are given in S2 Table.

### Starvation and differential feeding assays

The mosquitoes underwent several starvation durations. Starvation for 12 hours (s12), 24 hours (s24), 48 hours (s48) and 72 hours (s72). All of these groups were deprived of 10% glucose diet but given access to water ad libitum. A special starvation condition was utilized in the second differential feeding assay where mosquitoes were starved of 10% glucose as well as water for 12 hours (Star).

In the differential feeding assays, mosquitoes were trained under standard LD conditions for a week before changing the feeding conditions. The glucose control (GC) group was provided with a continuous 10% glucose meal. Blood meal-related groups which were the group that sampled immediately after blood meal (BF) and 24 hours post blood meal (PBM) (BFEXT) were provided with a blood meal. The reverse feeding (RF) group was provided with a sugar meal during the daytime and starved at night for 48 hours before being used in gene expression analysis, along with s24 group. In the second differential feeding assay, mosquitoes were provided with a Glucose meal (GC), Water (Wat), a Blood meal (BM), and completely starved (Star), respectively, before being used in gene expression analysis.

S24 and S48 groups were utilized in extended starvation experiments for gene expression analysis and all s12, s24, s48 and s72 were utilized in olfactory assay under extended starvation and periodic acid stain of fat bodies.

### Periodic acid stain

Mosquito fat bodies, along with the cuticle, were isolated from the mosquitoes and embedded in a paraffin block to make thin slices (1µM). The sliced mosquito fat bodies were then stained using periodic acid stain (PAS) (Wuhan Servicebio Technology Co. Ltd, CAT no. G1008) using a standard procedure to visualize glycogen deposits in the mosquito fat body. The fat bodies were observed under a microscope at 40X magnification.

### Dissection and assessing the blood digestion and reproduction health progress

12 hours post eclosion mosquitoes were divided into two groups and held together (both males and females) for one week under standard 12 hours light-dark cycles under standard conditions of mosquito rearing as mentioned earlier to ensure maximum likelihood of mating. After one week of training one group was provided with a restrained mouse for feeding at ZT4 under light conditions and second group at ZT16 under dark conditions. Both groups were given one hour to complete feeding. After one-hour mosquitoes were collected at 12 hours PBM, 24 hours PBM and 48 hours PBM and dissected on PBS to assess the blood bolus status in the midgut and the ovarian development. Ovarian development was measured using the ovarian length of each mosquito at each PBM timepoint. For each mosquito lengths were measured for both ovaries and average value was taken. To count the follicle number at 72 hours PBM, ovaries were obtained and the follicles were counted by taking photos and using Image J software. To obtain egg number we photographed eggs rafts laid and used the Image J counting function. Each egg raft was placed in separate bowl for hatching, once hatched the larvae was counted using the same method and hatching percentages were constructed using R using a mean egg count.

For RNA extraction for gene expressions, for trypsin genes midguts at each timepoint were obtained and the blood was washed using PBS before used in RNA extraction. For vitellogenin, fat bodies of the mosquitoes were used as tissue site.

### Statistical analysis

Experimental data were statistically analyzed using Real Statistics (Zaiontz, C. (n.d.). *Real Statistics Resource Pack*) in Excel (Microsoft Corporation, 2021). Relative gene expressions were calculated using 2^-ΔΔCt^ standard calculation procedure. All RTqPCR experiments contained three biological replicates. All the assays were done with two biological replicates. Intergroup comparisons were done using Student’s *t*-test. In case the assumption of equal variances between groups is violated *t*-test with unequal variance (Welch’s *t*-test variation) was utilized. Multigroup analysis was carried out using single-factor ANOVA followed by Dunnett’s post hoc test for comparing all groups to the control or Tukey post hoc analysis for all pairwise comparisons. For feeding assays, the data were analyzed using a generalized linear mixed model followed by ANOVA with Sidak correction. Olfactory assay data were analyzed using the chi-square test for independence. All assays were done in two replicates. PAS-stained area data were analyzed using the Kruskal-Wallis test, post hoc Mann-Whitney pairwise comparisons with Bonferroni correction. Statistical significance for all tests was determined at *P* < 0.05. All data are represented graphically in the format, Mean±SEM.

## Results

### *Culex pipiens* shows a distinctive feeding rhythm governed by an endogenous clock and entrained by light perception

**Fig 2:**
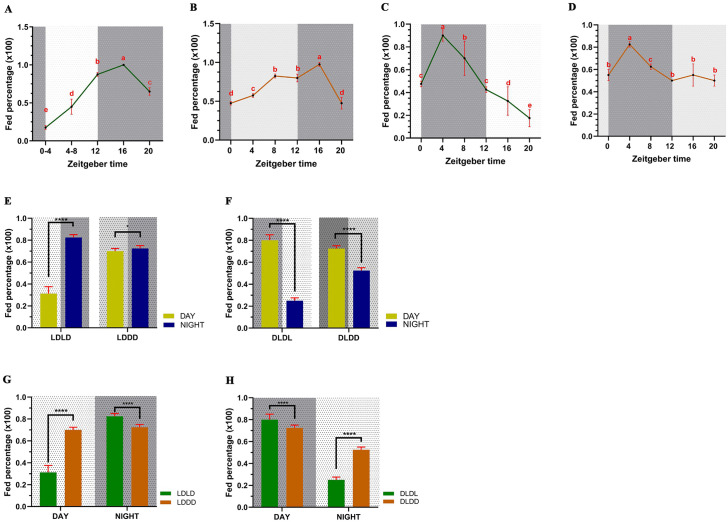
Feeding assay results. **A**- Feeding percentages measured under conventional 12h, light and dark cycle (LDLD). **B**- Feeding percentages under constant darkness after conventional light training (LDDD). **C**- Feeding percentages under reversed lighting conditions (DLDL). **D**- Feeding percentages under constant darkness after reversed light conditions (DLDD). Letters indicate statistical significance between each group (*P* < 0.005) (n = 20, per group). **E**- Total fed percentage comparison between daytime and subjective daytime and night and subjective nighttime under LDLD and LDDD conditions. **F**- Total fed percentage comparison between day and subjective day time and night and subjective night time under reversed DLDL and DLDD conditions. **G**- Fed comparison between LDLD and LDDD conditions under day and nighttime. **H**- Fed comparison between DLDL and DLDD conditions under day and nighttime. Readings from transition timepoints (ZT0 and ZT12) were excluded when calculating total fed percentages (n = 40, per group). Background represents the lighting conditions provided. White -Lights on period, Dark grey - Lights off period, Light grey - Lights off period correlates to subjective lights on period under LDLD or DLDL conditions. Statistical analysis was performed through a generalized linear mixed model followed by ANOVA with Sidak adjustment with two replicates, * *P* < 0.05, **** *P* < 0.0001. Data is represented as mean ± SEM.

Feeding assays were conducted using mosquitoes that were trained under different light regimes. Under diel conditions, *C. pipiens* displayed a clear nocturnal rhythm in blood-feeding (S1A and S1B Fig). Detailed temporal activity measurement under LDLD conditions, the feeding activity showed to be affected by the time of the day (GLM: *X*^*2*^ = 1797.3, df = 4, *P <* 0.001) with minimal feeding activity during the photophase, with gradually increasing activity towards the scotophase leading to a peak activity at ZT16 (Fig 2A). The rhythm was consistent in constant dark conditions in LDDD regime (GLM: *X*^*2*^ = 929.33, df = 5, *P* < 0.001) with the same peak activity time (Fig 2B), which indicates involvement of an endogenous circadian oscillator in maintaining the blood feeding rhythm.

Reversing the light conditions (DLDL/DLDD) induced a corresponding 12-hour shift in peak feeding activity to ZT4 (Fig 2C). This shift, which was also observed in constant darkness (DLDD; Fig 2D), collectively demonstrates that the rhythm is entrained by light perception. Satisfying two hallmarks of an endogenous rhythm, the data indicate a robust endogenous control that is entrained by light cues governing the blood feeding rhythm in *C. pipiens*.

### *Culex pipiens* feeding rhythm shows a masking effect through light perception

After excluding data from the transition periods (ZT0 and ZT12), analysis of the pooled data showed that a nocturnal feeding rhythm was maintained regardless of the lighting conditions, with peak feeding rates invariably confined to the dark phase. The consistency of the rhythm further confirms the endogenous control over the blood feeding rhythm. Differences in feeding rates were highly significant except in the LDDD regime. (Fig 2E and 2F) (GLM: all *P* < 0.001). A comparatively low significance was observed in LDDD conditions between the mosquitoes fed in dark in subjective light conditions and subjective dark conditions (GLM: *P* = 0.02), and the feeding rates under subjective daytime was elevated, which indicates that light exerts a direct inhibitory effect on the feeding rates as the feeding rates increase when the light is removed as an external cue (Fig 2E).

Because the pooled day-night contrast in LDDD was reduced (Fig 2E), we next compared feeding propensity during the same clock time windows across lighting regimes, daytime (ZT4 + ZT8) vs. nighttime (ZT16 + ZT20) in LDLD, and the corresponding subjective day and subjective night windows at the same zeitgeber times under constant darkness after LD training (LDDD) (Fig 2G). We performed the same windowed comparison for the reversed-trained groups (DLDL vs DLDD) (Fig 2H). Feeding rates were significantly elevated under the subjective day period in both LDDD and DLDD conditions (GLM: all *P* < 0.001), while a slight decrease was observed under the subjective night period (GLM: all *P* < 0.001) (Fig 2G and 2H). This increase occurred exclusively during the subjective daytime, obscuring the underlying endogenous rhythm. We term this phenomenon the “light-induced masking effect,” as it indicates that light actively suppresses feeding behavior to conceal the endogenous rhythm.

### *C. pipiens* feeding rhythm correlates with the expression of short neuropeptide F (sNPF)

**Fig 3:**
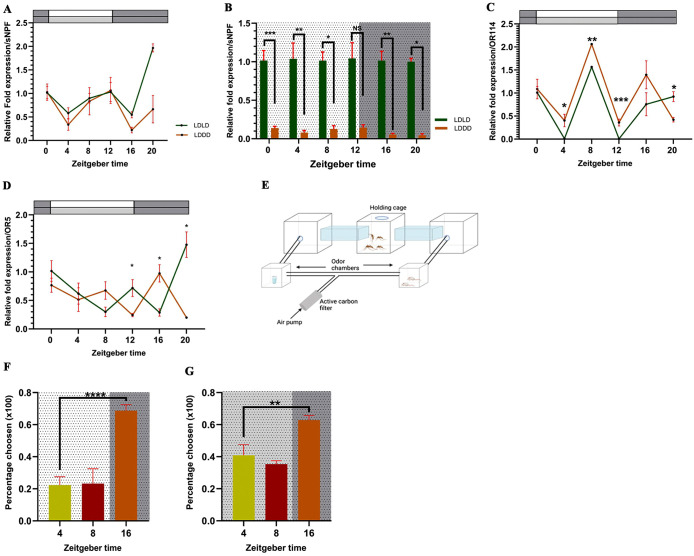
Endogenous expression of downstream neurotransmitter and olfactory response between LDLD and LDDD conditions. **A** -sNPF expression profile comparison between LDLD and LDDD. **B** - sNPF expression between LDLD and LDDD at individual time points. **C** - OR114 expression profile comparisons between LDLD and LDDD. Student’s *t*-test, *P < 0.05, **P < 0.01, ***P < 0.001. **D** - OR5 expression profile comparison between LDLD and LDDD. ANOVA single-factor, post hoc Dunnett’s test, *P < 0.05. The color bars at the top represent the lighting conditions: White - Lights on, Dark grey - Lights off, Light grey - Lights off period under LDDD conditions. **E** - Schematic representation of the three-cage olfactory assay. **F** - Olfactory response of mosquitoes under LDLD conditions. **G** - Olfactory response of mosquitoes under LDDD conditions. Chi-square of independence, **P < 0.01, ****P < 0.000, (n = 35-40, per group). Background color represents light conditions: White - Lights on, Dark grey - Lights off, Light grey - Lights off period under LDDD conditions. All data are represented as Mean±SEM. Fig 3E was drawn using Biorender (www.biorender.com).

Next, the role of the neurotransmitter sNPF was investigated. sNPF is a known modulator of the olfactory system in *Drosophila* [39] and a negative mediator of olfactory-driven host seeking in *Aedes spp* [40]*.* The expression profiles constructed for sNPF reveal a temporal oscillation in both conventional LDLD condition (ANOVA single-factor: *F* = 10.56, *r*^*2*^ = 0.81, *P* < 0.001) (S2A Fig) and in total darkness LDDD condition (ANOVA single-factor: *F* = 3.62, *r*^*2*^ = 0.6, *P* = 0.035) (S2B Fig). Post-hoc analysis using Dunnett’s test showed that under LDLD, sNPF downregulated at ZT16 compared to the control time point of 8.00 AM (D-test; $\bar{x}$_*diff*_ = -0.90, *P* = 0.040) (S2A Fig), aligning with the peak feeding time observed. Secondary downregulation at ZT4 was also observed, which was not significant (D-test; $\bar{x}$_*diff*_ = -0.84, *P* = 0.056) and unrelated to the feeding pattern, suggesting it might play a role in other physiological responses such as mating or sugar feeding, since sNPF is a neurotransmitter. A downregulation was also observed under LDDD conditions at ZT16 compared to the ZT0 control (D-test; $\bar{x}$_*diff*_ = -2.29, *P* = 0.026) (S2B Fig). The expression profiles of sNPF under both lighting regimes were closely aligned (Fig 3A), suggesting that sNPF expression is associated with the internal clock and may function as a output that corelates with the feeding rhythm.

Differential analysis confirms that under DD, sNPF expression is significantly lower than that of LD baseline. The time points of subjective daytime ZT0 (Student’s *t*-test; *t* = 9.92, df = 4, *P* < 0.001), ZT4 (Student’s *t*-*t*est; *t* = 6.38, df = 2.98, *P* = 0.007), and ZT8 (S*t*udent’s *t*-*t*est; *t* = 6.27,df = 2.52, *P* = 0.013) all showed downregula*t*ed sNPF expression while two time points at subjective night time, ZT16 (Student’s *t*-*t*est; *t* = 10.03, df = 2.84, *P* = 0.002) and ZT20 (S*t*udent’s *t*-*t*est; *t* = 6.49, df = 2.02, *P* = 0.022) was also significan*t*ly below the LD base line (Fig 3B). The differential expression of sNPF is consistent with the discrepancies in blood feeding rates observed between LD and DD, given that the existing data states sNPF signaling can suppress olfactory sensitivity in mosquitoes and thereby influence host seeking [40]. We also observed an upregulation of sNPF at ZT20 under LDLD (D-test; $\bar{x}$_*diff*_ = 0.97, *P* = 0.025), which indicates an anticipatory response to the oncoming photoperiod (S2A Fig).

### sNPF expression correlates with olfactory markers and blood feeding rhythm in *C. pipiens*

In exploring the role of the olfactory system in setting up *C. pipiens* blood feeding rhythm, a candidate olfactory receptor (OR114) was utilized. OR114 is a known receptor that is sensitive to the components of human sweat. Under the LDLD expression profile of OR114 showed temporal oscillation (ANOVA single-factor: *F* = 92.75, *r*^*2*^ = 0.99, *P* < 0.001) with an expression elevation at ZT16 (D-test; $\bar{x}$_*diff*_ = -0.48, *P* = 1.000), which correlates with the peak feeding. Interestingly, during the daytime point ZT4 and the transition time point of ZT12, expression of OR114 was close to zero, significantly dropping compared to the ZT0 control time point (D-test; $\bar{x}$_*diff*_ = -6.78, *P* < 0.001; $\bar{x}$_*diff*_ = -7.64, *P* < 0.001). Comparable to the sNPF expression profile, we observed a secondary, unrelated upregulation at ZT8 (D-test; $\bar{x}$_*diff*_ = 0.71, *P* = 1.000) (S2C Fig), which might hint at functions of OR114 other than the host seeking. Under LDDD conditions, OR114 manifested an elevated expression (S2D Fig). However, under both conditions, OR114 maintains the basic endogenous rhythm according to the visualization of the profiles (Fig 3C) and its concordance with the behavioral rhythm support an interpretation of endogenous regulation. We also observed that the LDDD profile is significantly higher in expression than the LDLD profile at ZT4 (Student’s *t*-test; *t* = 5.89, df = 1.74, *P* = 0.038), ZT8 (Student’s *t-*test; *t* = 85.53, df = 1, *P* = 0.007), and ZT12 (Student’s *t*-test; *t* = 14.69, df = 1.98, *P* = 0.004). Interestingly, we observed decreased sNPF expression and increased blood feeding propensi*t*y also in this time frame. Observed correlation of the blood-feeding rhythm with both OR114 and sNPF expression, together with prior evidence that sNPF inhibits olfactory sensitivity in mosquitoes [40], is indicative of a working model linking neuropeptide signaling, olfactory sensitivity and feeding propensity.

The olfactory assays confirmed that the secondary peaks that were observed are unrelated to the host-seeking mechanism of mosquitoes. Relative to the ZT4 baseline, olfactory response was minimal at ZT8 but pronounced at ZT16, with a readily observable increase in flight activity. This pattern of only high olfactory response at night time was consistent in both LD (*χ2* (1, *N* = 155)=33.03, *P* < 0.001) and DD groups (*χ2* (1, *N* = 150)=6.83, *P* = 0.008) (Fig 3F and 3G) compared to the ZT4 baseline and accompanied the initial hypothesis that feeding propensity and feeding time are separately controlled. The biological significance of the secondary oscillations in OR114 and sNPF profiles represents a fascinating area for future study, underscoringthe multi-layered complexity of mosquito chronobiology.

### In the absence of light, endogenous rhythm appears to take over diel rhythm in *C. pipiens* olfactory system

To gain a better understanding of the interactions between diel and endogenous rhythms, the expression profile of odorant receptor 5 (OR5) was created and analyzed, which is also expressed in the subterranean mosquito *C. molestus,* which is rarely exposed to normal LD cycles and might have a different rhythmic expression pattern from that of *C. pipiens,* therefore a different control mechanism. OR5 expression under LD and DD conditions showed two distinctive profiles (Fig 3D). In contrast to OR114, OR5 seemed to follow a diel expression profile, where under LD conditions OR5 expressed an inverse profile compared to OR114, but in the absence of light under DD, the expression pattern reversed to the endogenous expression pattern observed in OR114. Under LD, OR5 showed temporal oscillation (ANOVA single-factor; *F* = 10.91, *r*^*2*^ = 0.88, *P* = 0.003) with a significant downregulation at ZT16 (D-test; $\bar{x}$_*diff*_ = -1.53, *P* = 0.033), where the peak feeding time was observed. Another significant downregulation was observed at the ZT8 (D-test; $\bar{x}$_*diff*_ = -1.80,*P* = 0.015) (S2E Fig). OR5 under DD also showed evidence of transcription oscillation (ANOVA single-factor; *F* = 4.38, *r*^*2*^ = 0.65, *P* = 0.017) and followed a temporal expression pattern that is similar to that OR114’s bimodal endogenous profile (S2F Fig). The reversion of the rhythm was clearly evident in the dark period of the day where the LD expression was significantly different from the DD expression of OR5 at all time points of dark period, ZT12 (Student’s *t*-test; *t* = 4.20, df = 3.16, *P* = 0.022), ZT16 (Student’s *t*-test; *t* = 4.24, df = 3.48, *P* = 0.018) and ZT20 (Student’s *t*-test; *t* = 3.73, df = 2.66, *P* = 0.041). The shift of the profiles was attributed to the absence of light as an environmental cue under DD, demonstrating plasticity in olfactory regulation where underlying circadian rhythms compensate for the lack of environmental cues, given the critical nature of the olfactory system to the mosquito’s survival.

### Core clock gene CYCLE is responsible for feeding propensity control in mosquitoes

**Fig 4:**
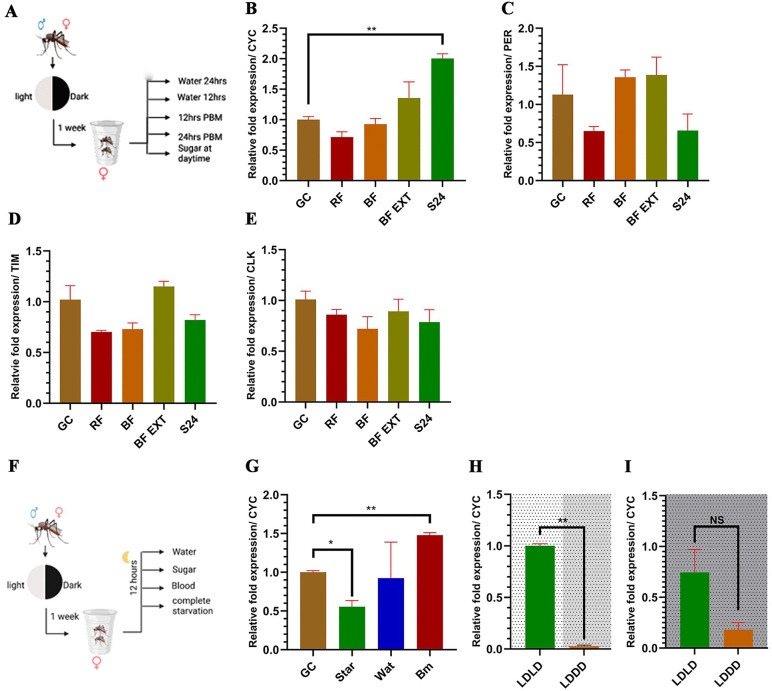
Core clock gene CYC is responsible for blood feeding propensity regulation. **A** - Experimental design for core clock gene expressions under different feeding conditions. **B** - CYC expression under different feeding conditions. **C** - PER expression under different feeding conditions. **D** - TIM expression under different feeding conditions. **E** - CLK expression under different feeding conditions. GC - Glucose control, RF - Reverse feeding conditions, BF - 12 hrs. post blood meal, BF EXT - 24 hrs. post blood meal, S24 - 24 hrs. starvation (with access to water), **F** - Experimental design for CYC expression vs different food sources. **G** - CYC expression under different food sources. GC - Glucose control, Star - 12 hrs. complete starvation (no access to water), BM - Immediately after blood meal, Wat - 12 hrs. water only diet. **H** - CYC expression in daytime between LDLD and LDDD conditions. **I** - CYC expression at night time between LDLD and LDDD conditions. Student’s *t*-test, *P < 0.05, **P < 0.01. All data are represented as Mean±SEM. Fig 4A and 4F were drawn using Biorender (www.biorender.com).

Differential feeding assays over five conditions revealed that only CYC responded among the canonical clock genes for the provided conditions (Fig 4A). Only feeding condition adjustment that was able to generate a significant response was starvation, which caused CYC to upregulate after 24 hours as evident by the results obtained compared to glucose control (Student’s *t*-test; *t* = 11.82, df = 2, *P* = 0.007) as a starvation induced response to metabolic stress. Because these comparisons were performed as feeding condition contrasts, they should be interpreted as temporal starvation-response effects rather than a rhythmicity analysis. Changing the feeding conditions, such as reverse feeding where a sugar meal during daytime and starvation at nighttime did not elicit a reaction in CYC, further suggesting the existence of temporal flexibility in mosquito feeding time windows. Interestingly, providing a blood meal during nighttime did not manage to elicit a response from CYC, 12 hours or after 24 hours PBM (Student’s *t*-test; *t* = 0.77, df = 2.64, *P* = 0.502; *t* = 1.39, df = 1.10, *P* = 0.381) (Fig 4B). Other clock genes (PER, CLK) did not respond to any of the conditions provided (Anova single-factor; *F* = 3.61, *r*^*2*^ = 0.61, *P* = 0.051; *F* = 1.12, *r*^*2*^ = 0.31, *P* = 0.400). While ANOVA indicate significance between groups in TIM, pairwise *t*-tests revealed that the significant was not among the groups and the control (ANOVA single-factor; F = 6.55, r^2^ = 0.72, *P* = 0.007) (Fig 4C–4E). Changing the food sources modulate the expression of CYC, in case of total starvation conditions, where mosquitoes were deprived of all food sources, including water, which caused the CYC to significantly downregulate (Student’s *t*-test; *t* = 6.31, df = 2.64, *P* = 0.011) and shortly after a blood meal to upregulate (Student’s *t*-test; *t* = 12.67, df = 2, *P* = 0.006) providing the first sign that CYC functions as a possible key mediator of metabolic state withing the clock mechanism for molecular stress modulate the feeding rhythm via integrating the information about mosquito internal environment conditions to the main TTFL. (Fig 4F and 4G).

CYC expression levels between LD and DD conditions were compared to establish a connection between the feeding rate differences observed during daytime and subjective daytime under LDLD and LDDD conditions. The CYC expression was significantly downregulated in subjective daytime under LDDD compared to LDLD (Student’s *t*-test; *t* = 11.36, df = 2.01, *P* = 0.008), with no significance at nighttime between the two lighting conditions, correlating with the observed phenomena of light-induced masking effect observed, establishing a link between feeding rates and CYC expression (Fig 4H and 4I).

### Time of the day blood feeding has a significant effect on blood digestion and reproduction in mosquitoes

**Fig 5:**
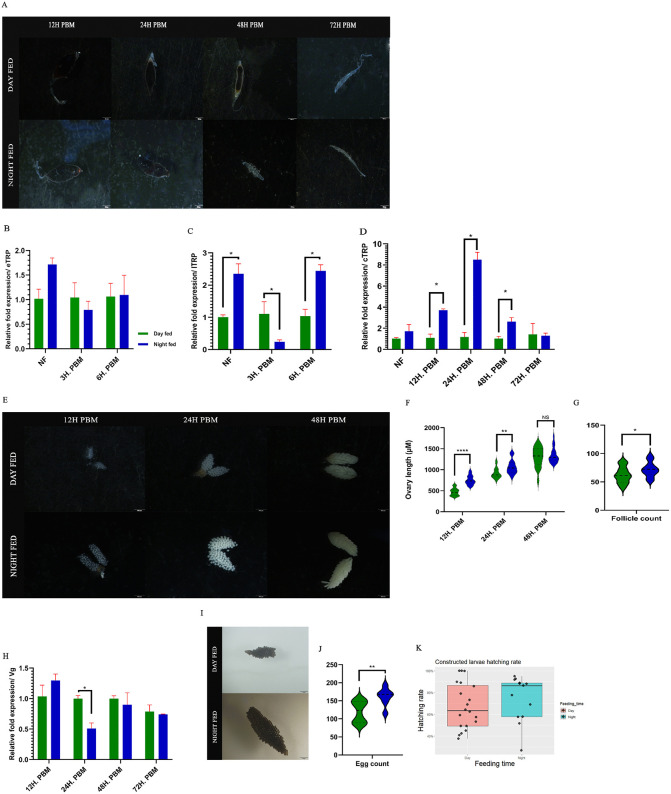
Mistimed blood feeding alters normal blood digestion and reproductive health. **A** - Comparison of the blood digestion state in the midgut between daytime and nighttime fed mosquitoes. **B** - Comparison of early trypsin (eTRP) expression in midgut between daytime and nighttime fed mosquitoes. **C** - Comparison of late trypsin (ITRP) expression in midgut between daytime and nighttime fed mosquitoes. **D** - Comparison of chymotrypsin (cTRP) expression in mosquito midguts between daytime and nighttime fed mosquitoes. **E** - Ovarian development comparison between daytime-fed and nighttime-fed mosquitoes. **F** - Ovary length comparison between daytime and nighttime-fed mosquitoes (n = 15-20, per group). **G** - Follicle count comparison between daytime and nighttime fed mosquitoes (n = 21, per group). **H** - Vitellogenin (Vg) expression comparison in mosquito fat body between day-time and night-time fed mosquitoes in PBM conditions. **I** - Egg raft comparison between daytime and nighttime-fed mosquitoes (n = 7-12, per group). **J** - Egg count per raft comparison between daytime and nighttime fed mosquitoes. Student’s *t*-test, *P < 0.05, **P < 0.01, ****P < 0.0001. **K** - Linear regression analysis results for the constructed hatch rate. The hatch rate was constructed using a mean egg count for each feeding time, and an arcsine-square root transformation to compensate. PBM - Post Blood Meal. NF - Non-blood-fed. All the scale bars represent 500μM. All data are represented as Mean±SEM.

To evaluate the impact of mistimed blood feeding, we assessed digestion and ovary development in day-fed (at ZT4) versus night-fed (at ZT16) mosquitoes. Visually, night-fed mosquitoes began digestion by approximately 12 hours PBM, whereas day-fed mosquitoes showed a significant delay. However, all mosquitoes completed digestion within 72 hours PBM (Fig 5A). This differential timing is likely explained by the day-night difference in late trypsin transcript abundance in non-fed mosquitoes and the sustained time dependent elevation of chymotrypsin observed post-blood meal (as detailed later). Exploring the genetic control of blood digestion-related enzymes, early trypsin and chymotrypsin showed no significant day-night difference at the sampled daytime and nighttime time points in the non-fed mosquitoes. Early trypsin expression between day-fed and night-fed mosquitoes remained comparable throughout the time points tested (Fig 5B). Interestingly, late trypsin showed a significant day-night difference at the sampled time in non-fed mosquitoes, where the night-time expression was significantly higher. (Student’s *t*-test; *t* = 5.71, df = 1.57, *P* = 0.049) and differentially expressed in the nighttime fed group at the PBM state compared to the daytime fed group at 3 hours PBM (Student’s *t*-test; *t* = 3.83, df = 4, *P* = 0.019) and 6 hours PBM (Student’s *t*-test; *t* = 4.04, df = 2.51, *P* = 0.038). Interestingly, at 3 hours PBM, a downregulation of late trypsin was observed, followed by an upregulation at 6 hours PBM. (Fig 5C). Chymotrypsin expression in night-fed mosquitoes was significantly higher compared to the day-fed mosquitoes in the PBM state at 12 hrs. (Student’s test; *t* = 4.28, df = 2.04, *P* = 0.048), 24 hrs. (Student’s *t*-test; *t* = 4.68, df = 2.05, *P* = 0.041) and 48 hrs. (Student’s *t*-test; *t* = 3.93, df = 4, *P* = 0.017 (Fig 5D). The higher expression of late trypsin in night time in non-fed mosquitoes and the higher expression of chymotrypsin in PBM state in night fed mosquitoes possibly contribute towards the delayed blood digestion observed in day-fed mosquitoes.

Significant reproductive consequences were observed as a result of mistimed blood feeding in the mosquitoes. Developmental delay in the ovaries, accompanied by diminished follicles per ovary and diminished egg count, was observed in the day-fed mosquitoes, where the night-fed mosquitoes showed early development in the ovaries at 12h PBM. The ovary length of night-fed mosquitoes was significantly larger up until 48h PBM, ovary length of the nighttime fed mosquitoes was significantly larger at 12 hours. PBM (Student’s *t*-test; *t* = 7.95, df = 31, *P* < 0.001) and 24 hours PBM (Student’s *t*-*t*est; *t* = 3.13, df = 37, *P* = 0.003). A*t* 48 hours PBM, ovarian development of day-fed mosquitoes caught up with the night-fed mosquitoes (Fig 5E and 5F). In PBM, Vitellogenin (Vg) plays a major role in ovary development by acting as a yolk protein precursor; therefore, Vg transcription was measured in the PBM state to explain how the ovary development of day-time fed mosquitoes caught up to the night-time fed mosquitoes. Vg expression data revealed that at 24 hours, PBM day-fed mosquitoes held a significantly higher Vg expression compared to the night-fed mosquitoes (Student’s *t*-*t*est; *t* = 6.98, df = 1.36, *P* = 0.049), where night-fed mosquitoes started reducing the expression, possibly as a compensation mechanism (Fig 5H). This was further evidenced by the expression profile over time in day-fed mosquitoes (ANOVA single factor; *F* = 29.59, *r*^*2*^ = 0.95, *P* = 0.001). Post hoc analysis (Tukey’s HSD) revealed distinct Vg expression dynamics between the groups. In daytime-fed mosquitoes, Vg expression remained stable between 12 and 24 hours PBM (*P* = 0.999, 95% CI [-1.07, 1.15]) and did not decline significantly until 48 hours PBM (*P* = 0.002, 95% CI [1.40, 3.84]). In contrast, nighttime-fed mosquitoes exhibited a dynamic Vg profile (ANOVA, F = 38.76, r² = 0.96, *P* < 0.001), with downregulation initiating significantly earlier, at 24 hours PBM (*P* = 0.015, 95% CI [0.45, 2.96]). Vg expression in both groups began to recover by 72 hours PBM. These opposing dynamics suggest the existence of a compensatory mechanism to mitigate the adverse effects of mistimed blood feeding. Despite the compensation, the day-fed mosquitoes contained a smaller number of follicles than the night-fed mosquitoes (Student’s *t*-test; *t* = 2.22, df = 40, *P* = 0.032) (Fig 5G). Correlating to this, the day-fed mosquitoes also laid a smaller number of eggs than the night-fed mosquitoes (Student’s *t*-test; *t* = 3.16, df = 17, *P* = 0.006) (Fig 5I and 5J), but the hatching rate between the groups remained similar according to the simple linear regression performed. The overall model was not significant (*F*(1,31)=0.35, *P* = 0.56). The model accounted for a negligible variance (Adjusted *r*^*2*^ = -0.021). The results indicated that feeding at night was unable to predict a change in the transformed hatch rate compared to the feeding at daytime (*β* = 0.058, SE = 0.099, *t*(31)=0.59, *P* = 0.56) (Fig 5K).

### Mosquitoes shut down the olfactory system as a response to extended starvation

**Fig 6:**
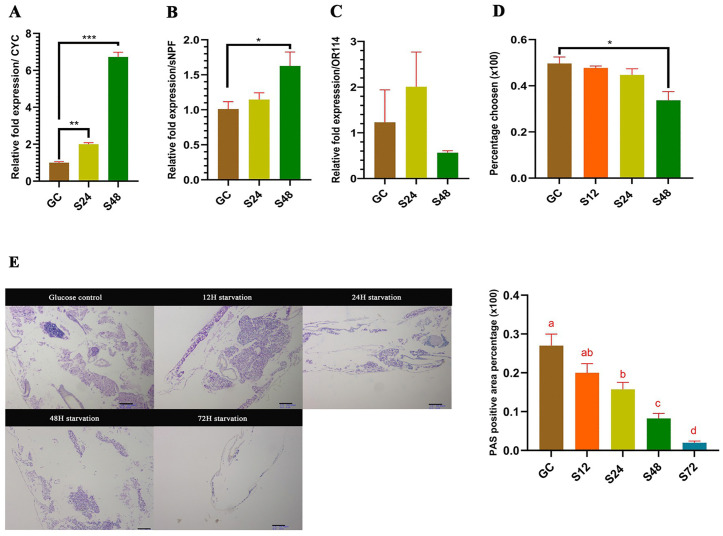
CYC-mediated shutdown of olfactory under metabolic stress. **A** - CYC response to extended starvation. **B** - sNPF response to extended starvation. **C** - OR114 response to extended starvation. GC - Glucose control, S24 - 24 hrs. water diet, S48 - 48 hrs. water diet. Student’s *t*-test, *P < 0.05, **P < 0.01, ***P < 0.001. **D** - Olfactory assay results for starvation conditions. S12 - 12 hrs. water diet. Chi. Square of indipendence (n = 32-40, per group), *P < 0.05. **E** - Periodic acid staining (PAS) of the fat body for glycogen deposits and areas positive for PAS in tissue. All scale bars represent 100uM. Statistical analysis was done through the Kruskal-Wallis test (n = 4, per group), followed by Mann-Whitney pairwise comparisons with Bonferroni correction. Letters indicate the significance between groups. All data are represented as Mean±SEM.

To assess the metabolic response in mosquitoes to prolonged nutrient deprivation, the CYC response to the extended starvation was investigated. The expression of CYC increased significantly as the starvation progressed to 24 hours starvation (Student’s *t*-test; *t* = 11.82, df = 2, *P* = 0.007) and 48 hours starvation (Student’s *t*-test; *t* = 31.99, df = 2, *P* < 0.001) compared to a control group which received a continuous 10% glucose supply. We design our experiment to test starvation duration effects, not to evaluate circadian rhythmicity across the day. This phenomenon was only seen in CYC and not in other canonical clock genes (Figs 6A and S3). The CYC upregulation was correlated with significant upregulation of sNPF at 48h starvation compared to a control group with continuous 10% glucose supply (Student’s *t*-*t*est; *t* = 3.03, df = 4, *P* = 0.039) (Fig 6B). No significan*t* upregulation in sNPF was observed at 24 hours starvation point, but the overall graph showed an increasing trend in expression as the starvation progressed. While high variance reduced statistical power for individual comparisons by *t*-*t*est, a significant overall effect was detected by ANOVA (ANOVA single-factor; *F* = 5.13, *r*^*2*^ = 0.72, *P* = 0.043), and a consistent downregulation trend was observed. The post-hoc Tukey’s HSD test found that compared to 12 hrs. starvation at 48 hrs. starvation, OR114 is significantly downregulated (*P* = 0.041, 95% CI[0.09,3.86]) confirming that the OR114 is also downregulated as the starvation progress. This was further confirmed through olfactory assay, which showed a significant reduction of olfactory response at 48 h starvation (*χ2* (1, *N* = 152)=4.12, *P* = 0.042) (Fig 6C and 6D). Furthermore, this downregulation occurs in parallel with a significant depletion of glycogen deposits in mosquitoes’ fat bodies. Glycogen is the cellular energy reserve in mosquitoes, and from 48h to 72h of starvation, a significant reduction in glycogen deposits was identified in the fat body across the starvation points using the Kruskal-Wallis H test to compare the area stained in the fat body tissue by PAS (*χ2*(4)= 17.24, *P* = 0.001).Post-hoc Mann-Whitney comparisons revealed a progressive depletion of glycogen reserves, which began at 24h of starvation (Mdn = 0.15, *P* = 0.030), continued through 48h (Mdn = 0.08, *P* = 0.030), and persisted up to 72h.starvation (Mdn = 0.02, *P* = 0.029), where the mosquitoes started dying. This depletion pattern corresponds with the CYC upregulation that was observed previously (Fig 6E), and sNPF upregulation pattern with the OR114 expression pattern, along with the olfactory assay data, suggest that *C. pipiens* downregulates high-energy-demanding systems, such as olfactory, during metabolic stress, alongside CYC upregulation, consistent with a stress-responsive interaction between metabolic state and circadian regulation.

### CYC knockdown alters day vs. night expression differences in canonical clock genes and affects sNPF expression

**Fig 7:**
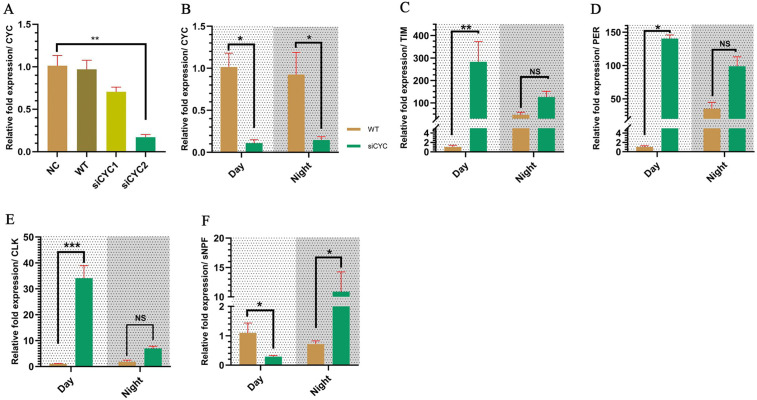
Loss of function of CYC disrupts the day-night differences in core clock expression and alters sNPF expression. **A** - siRNA efficiency against CYC. siCYC2 showed around 83% efficiency and will be referred to as siCYC from here on. **B** - CYC expression day vs night between WT and siCYC. **C** - TIM expression day vs night between WT and siCYC. **D** - PER expression day vs night between WT and siCYC. **E** - CLK expression day vs night between WT and siCYC. **F** - sNPF expression day vs night between WT and siCYC. All the expression was measured at two anchor points ZT4 and ZT16. Student’s *t*-test, *P < 0.05, **P < 0.01, ***P < 0.001. All data are represented as Mean±SEM. NC - Negative control, WT - Wild type, siCYC1 - RNA interference group 1, siCYC2 - RNA interference group 2.

To solidify the link between CYC and blood-feeding rhythm, expression of CYC was knocked down using siRNA. The sequence used managed to knock down the CYC expression with 83% efficiency at day three post-injection (Student’s *t*-test; *t* = 8.26, df = 3.30, *P* = 0.002) (Fig 7A). For this analysis, gene expression was measured at one representative daytime and one nighttime anchor time points (ZT4 and ZT16) at mid photophase and mid scotophase to assess day vs. night contrasts but a full 24-hour profile would be required to formally demonstrate oscillations and quantify rhythmic parameters. This altered day - night expression differences of all the clock genes, specifically in daytime expression. Among them, PER (Student’s *t*-test; *t* = 18.79, df = 1.05, *P* = 0.030) and TIM (Student’s *t*-test; *t* = 11.95, df = 1.99, *P* = 0.006) showed highly elevated daytime expression compared to wild type, likely due to the disrupted negative TTFL. CLK also exhibited an elevated daytime expression compared to wild type (Student’s *t*-test; *t* = 15.78, df = 2.92, *P* < 0.001). High expression of all canonical clock genes of the TTFL in daytime is indicative of a disrupted core clock state at the sampled daytime anchor (Fig 7B–7E). Further analysis of TIM using ANOVA (ANOVA single-factor; *F* = 76.44, *r*^*2*^ = 0.98, *P* < 0.001) showed that the day-night expression was also changed. Tukey HSD test showed that in wild type mosquitoes daytime expression of TIM was lower than the nighttime (*P* = 0.002, 95%CI[3.15,7.87]), but when CYC expression was knocked down, the two groups lost significant difference (*P* = 0.342, 95%CI[-1.23,3.48]) (S4A Fig). The same analysis was done for PER (ANOVA single-factor; *F* = 163.90, *r*^*2*^ = 0.99, *P* < 0.001). PER expression in wild type mosquitoes, nighttime expression was higher than the daytime (*P* < 0.001, 95%CI[3.71,6.47]), but in the CYC knockdown group, the difference between them was lost (*P* = 0.494, 95%CI[-0.73,1.78]) (S4B Fig).The CLK gene showed significant in expression at daytime in siCYC group (*P* = 0.017, 95%CI[0.53,4.03]) and CYC showed no significance in either group (S4C and S4D Fig). All of these results are indicative of a dysfunctional core clock mechanism when assessed as a day - night contrast at the sampled timepoints.

A key finding concerns sNPF, a well-established negative regulator of mosquito olfaction [40]. Its day-night expression difference at the sampled time points was significantly altered in siCYC mosquitoes compared to the wild-type controls. Specifically, reversal of the day-night contrast relative to WT was observed in sNPF when CYC expression is knocked down, where daytime expression was lower compared to the wild type mosquitoes (Student’s *t*-test; *t* = 3.76, df = 2.71, *P* = 0.039) and nighttime expression was higher compared to the wild type (Student’s *t*-test; *t* = 7.66, df = 1.41, *P* = 0.041) (Fig 7F). We also observed a night biased expression between the two points tested in siCYC group (S4E Fig). Therefore, unlike in wild-type mosquitoes, siCYC mosquitoes are predicted to exhibit a greater blood-feeding propensity during the day than at night based on these day-night differences measured at the sampled time points.

### CYC adjusts the feeding propensity of the mosquitoes but not the feeding time window

**Fig 8:**
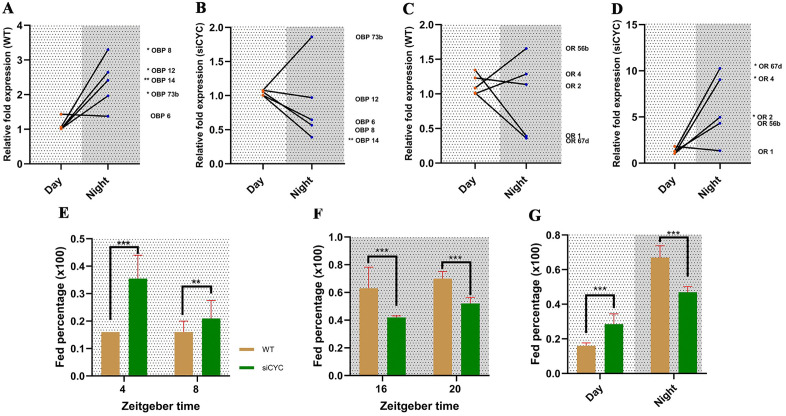
Silence of CYC alters Olfactory-related gene day-night expression contrasts and alters blood feeding propensity. **A** - Odorant binding protein day-night expression contrasts in wild-type mosquitoes. **B** - Odorant binding protein day-night expression contrasts in the siCYC group. **C** - Odorant receptor day-night expression contrasts in wild-type mosquitoes. **D** - Odorant receptor day-night expression contrasts in the siCYC group. All expression data was measured at two anchor points (ZT4 and ZT16). Student’s *t*-test, *P < 0.05, **P < 0.01. **E** - Blood feeding propensity in daytime between the wild type and siCYC group. **F** - Blood feeding propensity at night time between wildtype and siCYC group. Feeding propensity in E-F was assayed at the same non-transition time points used in Fig 2. **G** - Compiled blood feeding propensity differences between day and nighttime between wild-type and siCYC groups. Mosquito whole heads with the antenna was utilized as the tissue site, where the siRNA efficiency was already validated. Statistical analysis through generalized linear mixed model followed by ANOVA with Sidak adjustment (n = 13-25, per group), **P < 0.01, ***P < 0.001. All data are represented as Mean±SEM.

To visualize the behaviour of the olfactory system in the absence of CYC expression, five OBPs genes and five OR genes were randomly selected to compare the expression between day and nighttime in both groups of WT and siCYC. The majority of OBPs (four out of five tested) showed significant upregulation at night time in wild-type mosquitoes, indicating a heightened olfactory activity at night time, which facilitates locating suitable hosts for blood feeding (Fig 8A). This nocturnal pattern was abolished in the siCYC group, with several genes losing significance (three out of five tested). OBP14 showed a significant downregulation at night compared to the daytime in the siCYC group (Student’s *t*-test; t = 83.96, df = 1.41, *P* = 0.001). The OBP gene 73b was considered as an outlier in the siCYC group (Fig 8B). The data suggest that in the wild type mosquitoes, olfactory sensitivity was high at night time, facilitating host seeking and thereby increasing blood feeding propensity at night time, while siCYC group has a comparable olfactory sensitivity all around or less sensitivity at night time, thereby attenuating the day-night contrast in feeding propensity at the tested time points.

In contrast to the wild type, which showed no clear diurnal rhythm in OR expression, the siCYC group exhibited a dysregulated nocturnal pattern (Fig 8C and 8D). Further analysis revealed that the knockdown of CYC alters the baseline expression of the majority of OBPs in all conditions and all the ORs in all conditions (S5 Fig), indicating that CYC plays a major role in controlling the overall tone of the mosquito olfactory system. The consequences of CYC knockdown on blood-feeding propensity were significant. To enable direct comparison with the wild-type feeding profile in Fig 2, we assayed feeding propensity at four time points that overlap with those previous results and exclude transition periods. siCYC mosquitoes showed a significant increase in blood feeding propensity in daytime compared to wild type at both time points tested, ZT4 (GLM; *P* < 0.001) and ZT8 (GLM; *P* < 0.001), and a significantly lower feeding propensity at nighttime compared to WT mosquitoes at the two time points tested, ZT16 (GLM; *P* < 0.001) and ZT20 (GLM; *P* < 0.001) (Fig 8E and 8F). This indicates a shift in blood feeding propensity towards daytime in siCYC groups. However, despite the alterations in feeding propensity, compiled analysis revealed that the shutdown of CYC expression cannot completely demolish the endogenous rhythm. The wild-type mosquitoes and siCYC group both retained their overall nocturnal feeding rhythm (GLM; all, *P* < 0.001). Because of the close nature of feeding propensity between day and night odds ratio was also calculated to further validate the results in the siCYC group, which showed that the difference observed was robust (Odds ratio = 0.53, *P* < 0.001). Alteration of feeding propensity between day and night and the inability to alter the time window of overall blood feeding rhythm even in the absence of proper functioning core clock due to the loss of function of CYC in siCYC mosquitoes, reveals remarkable system robustness, suggesting the existence of compensatory mechanisms such as involvement of CRY, existence and involvement of local clock mechanisms or redundant timing pathways in regulating mosquito blood feeding behaviour. (Fig 8G).

### CYC knockdown alters the preparation for blood digestion in mosquitoes

**Fig 9:**
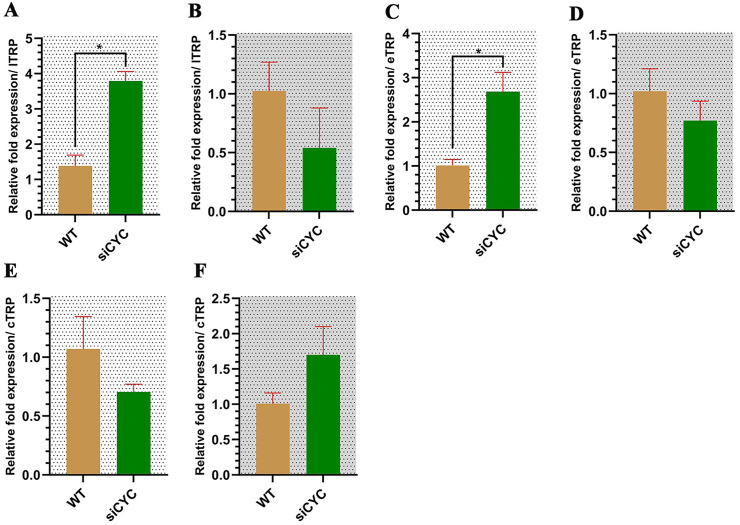
Loss of CYC alters the expression of trypsin transcription. **A** - Late trypsin (1TRP) expression. **B** - Late trypsin expression at night. **C** - Early trypsin (eTRP) expression in daytime. **D** - Early trypsin expression at night. **E** - Chymotrypsin (cTRP) expression in daytime. **F** - Chymotrypsin expression at night. Student’s *t*-test, *P < 0.05. All data are represented as Mean±SEM.

Finally, we checked the expression of the genes related to blood digestion in mosquitoes in response to the CYC knockdown. In relation to late trypsin expression, we observed that the late trypsin expression is significantly higher in the siCYC group during daytime (Student’s *t*-test; *t* = 5.18, df = 2.15, *P* = 0.030) and showed no significant difference at nighttime (Fig 9A and 9B). eTRP expression was also significantly elevated during day daytime in siCYC group compared to the WT (Student’s *t*-test; *t* = 4.53, df = 1.95, *P* = 0.048) but not at night. (Fig 9C and 9D). Chymotrypsin expression showed no significant differences between the two groups at either daytime or nighttime (Fig 9E and 9F).This is also interesting as in our previous experiments, we determined that the late trypsin is the only blood digestion-related gene that seemed to be differently expressed between day and night, and therefore the best candidate to express a temporal expression pattern, but loss of function of CYC also caused early trypsin to upregulate in daytime compared to wild type. The upregulation of late trypsin and early trypsin in the daytime in the siCYC mosquito group can be interpreted as a result of dysregulation of the temporal coordination of blood digestion genes following core clock disruption, which may coincidentally facilitate blood digestion if a blood meal is acquired at this non-typical time.

## Discussion

Our comprehensive investigation reveals that the blood-feeding rhythm in *C. pipiens* is driven by a robust endogenous clock that persists in constant darkness. Furthermore, we have proposed CYCLE-sNPF-olfactory axis as a candidate pathway regulating feeding propensity. Crucially, this axis is modulated by the organism’s metabolic state, with CYCLE acting as the key mediator. Mistimed blood feeding has a severe physiological impact on mosquito reproduction, emphasizing the importance of a core clock that regulates correct feeding timing. Furthermore, CYC knockdown was only able to alter the feeding propensity, not the timing, indicating that there might be multi-layered control in the rhythm and overall resilience of the system. The clear separation of stable time dependent window for blood feeding and feeding propensity that can be tuned independently aligns with the broader chronobiology frameworks that was already observed in which the internal circadian clock set the optimum phases in the time of the day while neuromodulators and the physiological state of the organism adjusts the behavioural intensity of the particular rhythmic activity [41] The layered control we propose is also evident by the QTL of *C. pipiens* that did not contain the canonical clock genes that the feeding time may be controlled from outside the TTFL [42].

Importantly, similar clock-physiology connections have been reported in other mosquito vectors, supporting the generalize relevance of the model we propose. In *Aedes aegypti*, blood feeding and insemination can alter the transcription of core clock genes, consistent with bidirectional relationship between the post blood meal physiology and the circadian system [43]. Moreover, disruption of circadian function in Ae. aegypti impacts multiple fitness and behaviour linked traits, indicating that clock components can affect the functions of downstream outputs rather than acting as passive correlates [44]. Comparative work further shows that the relative effects of environmental inputs differs within species such as *A. aegypti* and *Culex quinquefasciatus* that display distinct activity rhythms and clock gene expression to light versus temperature [34] suggesting a conserved circadian model tuned by species specific environmental limitations.

In the absence of light, the rhythm remains strongly nocturnal, disregarding whether the external cues are present or not after successful training. The reverse of light conditions during the training period reversed the feeding rhythm, indicating that the overall rhythm is sensitive and can be trained using the external environmental cues. The observed characteristics of the blood-feeding rhythm-its free-running nature in constant darkness and its acute suppression by light (the ‘light-induced masking effect’) are consistent with endogenous circadian control. This phase and amplitude separation pattern are consistent with the broader chronobiology models where the entrainment sets phase while masking superimposes state dependent intensity inputs without needing to completely reset the established clock while acting as two different systems [7,45]. Furthermore, the effect of light and the feeding propensity has been dissociated experimentally in *Anopheles spp.* where the short light pulses suppress the feeding in clock independent manner while longer light exposure suppress the feeding in clock dependent manner which are consistent with the distinctions between the masking and entrainment conditions [46]. Comparable data has been revealed in recent years with other mosquito species specifically with *Anopheles spp.* where it has been demonstrated that the biting rhythms can persist in the absence of light input white acute light exposure can suppress the biting activity in the nocturnal stage of the day, which has been speculated as a potential vector control strategy in *Anopheles* mosquitoes [47,48] while the acute exposure of light at night increase the biting preference in the diurnal mosquito *Aedes aegypti* [37]indicating that the modulatory effect of light is depend on the circadian niche of the organism and support idea of the light input routing in to opposite behavioural outputs [49]. Furthermore, the differential expression of CYC between light and dark conditions during the subjective day suggests its involvement in this phenomenon. While the precise mechanism requires further elucidation, the demonstrated role of light implicates circadian photoreceptors, with CRY representing a plausible candidate for mediating this masking effect. Recent work into the rhythmic behaviors of Aedes albopictus and Culex quinquefasciatus provide evidence of photoreceptive pathways including opsins and importantly cryptochrome 1 (CRY1) that contribute to light mediated regulation of circadian rhythms that solidify the possibility of the existence of CRY1 mediated or linked masking or entrainment mechanisms in mosquitoes. These observations suggest that these conserved photo input components may contribute to the entrainment and masking effects across the mosquito species [50]. This highlights the importance of ambient light not only as a cue to establish the endogenous rhythm but also as an acute suppressor of mosquito blood feeding behaviour.

Our data indicate that the sNPF express time of day variations that corelates with the olfactory marker and the feeding propensity observed, with its rhythm being dependent on CYC function as these variations was altered when CYC translation was inhibited. Given that the previous data indicate sNPF can inhibit olfactory sensitivity in insects this pattern is consistent with an sNPF mediated olfactory gated model. Since our study did not silence sNPF directly we present this evidence as corelative. Olfactory maintains the host-seeking nature of mosquitoes and is therefore important for maintaining the blood-feeding rhythm. The inverse correlation between sNPF expression and blood-feeding rates is consistent with sNPF acts as an inhibitory signal that link clock output to olfactory system, consistent with the possibility that circadian signals contribute to time-of-day differences in olfactory sensitivity. Accordingly, the sNPF expression profile inversely matched that of OR114, supporting a model in which rhythmic sNPF may contribute to the variations in the olfactory sensitivity according to the time of the day. The notion of the presence of a neuropeptide gate in the control of rhythmic behaviors such as food sourcing is well studied in the *Drosophila* where the sNPF signaling in the olfactory system that modulate the sensitivity of the system coupling with the Insulin signaling that generate as a result of starvation, which demonstrate a link between the sNPF transcription, olfactory regulation and the metabolic state [39]. It has been demonstrated that the neuromodulation contributes to the host-seeking drive in the mosquitoes, particularly in *Aedes spp*. it has been demonstrated that the host seeking regulation post blood meal is regulated thorough NPY- like receptor pathways, which further validate our current sNPF-olfactory control architecture [51]. Notably, functional evidence in *A. aegypti* supports the likelihood of an inhibitory neuropeptide gate presence upstream of host seeking. Changes in sNPF combined with allatostatin-A within antennal lobes reduce odor mediated host seeking, and stimulation of these peptides suppresses host-seeking behavior [40,39]. Therefore, while our proposed CYC -sNPF linkage in *C. pipiens* is based on expression patterns and is presented here as an interpretation, the generality of sNPF-associated inhibition of olfactory-driven host seeking has been demonstrated in major vector species, strengthening the cross-species implications for our model. Our subsequent experiments identified that among the core clock genes, CYC was uniquely responsive to metabolic stress conditions. The specific role of CYC, rather than its heterodimeric partner CLK, as the primary metabolic responder is intriguing and suggests a potential specialization within the TTFL for energy sensing. It has been described well that the core time keeping and the behavioural outputs in other insect species, where the clock coordinates the feeding and metabolism, while peripheral and central clocks can differentially contribute to metabolic pathways, allowing flexible control in intensity while maintaining robust timing [52]. We further confirmed this by measuring the presence of glycogen in the fat body and comparing it to the shutdown of the olfactory system we observed. Glycogen reserves were significantly depleted after 24h of starvation, coinciding with the initial upregulation of CYC. This was followed by a subsequent increase in sNPF at 48h, suggesting a temporal sequential activation wherein CYC and responds to metabolic stress, which is followed by downstream increased sNPF transcription, which may result in the olfactory shutdown we observed. Collectively, our data supports a CYC-sNPF-olfaction model that suppresses olfactory activity to conserve energy during metabolic stress and at circadian-inappropriate times. In the diapause state, the *C. pipiens* exhibits major shifts in feeding strategies, prioritizing carbohydrate accumulation rather than the blood feeding, indicating that the metabolic states can severely affect the feeding patterns within *C. pipiens* [53].

In the case of the olfactory system, it showed a complex regulation. The candidate gene OR114 showed a rhythm that is endogenous in nature and aligns with the feeding rhythm observed. The OR5 expression profile showed a pattern which is a total inversion of that we observed for OR114 under LD condition which led us to believe that it is under diel rhythm but interestingly once the light cue was removed the rhythm instead of total abolish reverted to the overall endogenous profile in DD which highlights the plasticity of olfactory system where the underlying rhythms can compensate for absence of environmental cues. These clock driven rhythmicity in the olfactory system are widely reported in other mosquito genera. Multiple odorant-related genes and proteins show daily oscillations in *Anopheles spp.* that coincide with the nocturnal peak in the blood feeding [54]. The same rhythmic behaviour in odorant sensitivity in the *Aedes spp.* has also been reported [55] which supports our claim that the odorant sensitivity biases the host seeking towards the circadian window of blood feeding.

siRNA-mediated knockdown of CYC confirmed its pivotal role: it disrupted the day-night expression states of core clock genes and inverted the day-night transcriptional state of sNPF at the anchor points. This inversion in siCYC mosquitoes was followed by increased daytime and a decrease in nighttime blood-feeding propensity, which is consistent with an altered clock impacting the downstream pathways that rearrange the feeding intensity. We attribute the lack of a significant day-night difference in sNPF expression in wild-type mosquitoes to our sampling times (ZT4, ZT16), which coincided with the troughs of its expression profile. Together, the disrupted sNPF rhythm and the corresponding shift in feeding behavior solidify the role of CYC as a central metabolic integrator within the circadian system that modulates blood-feeding rhythm. However, the overall feeding rhythm maintained a nocturnal behaviour even in the absence of CYC function. The persistence of overall nocturnal rhythm suggests that CYC primarily adjusts the intensity of blood feeding rather than the temporal window itself. This indicates a decoupling between time window and feeding rate, highlighting the robust nature of the circadian system, accompanied by compensation mechanisms that safeguard essential behaviours like host seeking. Maintaining the overall temporal activity framework, isolated from the mechanisms that modulate the feeding rates, ensures the survival and function of vital systems in case of disturbance in the core clock or stress conditions. The work on identifying CYC targets in the Culex genome provides insight into why partial disturbance of the clock could only affect the selected downstream pathways while leaving the other timing-related pathways unaffected, furthermore revealing that the circadian window selecting pathways can be separated from the clock-controlled sensory modulation, which is consistent with the layered control system of blood feeding rhythm we propose here [49].

Our study further reveals that mistimed blood feeding severely compromises reproductive fitness in *C. pipiens*. Daytime feeding led to delayed blood digestion and consequently impaired ovarian development and egg production, underscoring the critical importance of circadian timing for mosquito fitness. This digestive delay is consistent with the rhythmic expression of key enzymes; for instance, late trypsin, which is poised for high expression at night, and chymotrypsin, which sustained higher expression in night-fed mosquitoes. The observed impairment in ovarian development is therefore consistent with the reduced blood digestion efficiency in the daytime fed mosquitoes. Notably, we observed a prolonged vitellogenin (Vg) expression in daytime fed mosquitoes that appears to be a compensatory response to mitigate the adverse effects and sustain reproduction, indicating a remarkable plasticity in the blood-feeding system. Interestingly, knockdown of CYC dysregulated the daytime expression of digestive enzymes. This indicates that the circadian control of feeding rates, which ensures feeding occurs at the optimal, predetermined time is coordinated with the expression of blood digestion enzymes. CYC knockdown disrupts the coordination between these two processes, which suggests that the CYC or the clock output is coupled with digestive preparedness, although the mechanistic connections are yet to be confirmed. Consequently, misalignment between feeding time and digestive readiness imposes severe reproductive costs. These findings are backed by the previous work that indicates that the blood intake can itself feedback to the circadian clock-regulated genes in mosquitoes, indicating a bidirectional relationship between the feeding and the time-keeping mechanism, specifically in the *Aedes aegypti* where the act of blood ingestion has a direct impact on the clock genes [43,44]. Furthermore, disruption of the circadian clock alters both feeding behaviour and reproductive fitness in *Aedes spp.* further strengthening the interpretation that the correct alignment of feeding rhythm and reproductive fitness may extend across the mosquito taxa [44].

Overall, our findings suggest that in the circadian system CYCLE is uniquely responsive to the metabolic state within the mosquito and consistent with a model mechanism that modulates blood-feeding intensity through the sNPF-olfactory pathway gate, which we propose based on the established data on mosquitoes and our expression-phenotype data, while leaving the core timekeeping mechanism intact. To our knowledge, our study is the first to provide evidence on the presence of a molecular link between metabolic sensing, olfactory control, and feeding behavior in *Culex pipiens*. The identification of CYC as a novel regulator opens new avenues for vector control. Specifically, targeting the CYC-CLK heterodimer or disrupting CYC’s ability to mediate metabolic stress could desynchronize mosquito biting propensity from human activity patterns. Such a strategy would reduce mosquito-host contact and potentially diminish disease transmission. Previous work demonstrating the use of light to modulate the biting behaviour of the nocturnal feeding mosquitoes and possible pharmacological interventions targeting the neuropeptide pathways provide a potential route to separate feeding propensity from the peak circadian window [48,51].

## Conclusion

Our investigation indicates that the blood-feeding rhythms of *Culex pipiens* are consistent with regulation by an endogenous pacemaker, entrained primarily by light. We identified using our data and established sNPF liked olfactory modulation described in other insects that the core clock gene CYCLE as a principal regulator of feeding rate, and that the rhythmic sNPF expression could represent a link between the circadian timing information and olfactory sensitivity, a key determinant of host-seeking behavior. While CYC knockdown disrupted core clock rhythmicity and inverted sNPF expression, shifting feeding propensity toward the daytime, it did not abolish the fundamental nocturnal rhythm, suggesting the existence of compensatory or locally functioning clock mechanisms.

We further showed that mistimed blood feeding carries severe fitness costs, impairing blood digestion, delaying ovarian development, and reducing fecundity. These findings underscore the adaptive significance of temporal alignment between feeding and internal physiology. Moreover, CYC upregulation during metabolic stress, coupled with subsequent sNPF induction and olfactory shutdown, reveals an energy-saving mechanism that curtails host-seeking when internal resources are depleted (S6 Fig).

The identification of CYC as a nexus of circadian timing and metabolic sensing reveals a potential vulnerability in mosquito chronobiology. Targeting the CYC pathway, for example, through small-molecule inhibitors that disrupt its interaction with CLK or its stress-sensing function, represents a promising strategy for vector control. Such interventions could desynchronize mosquito biting activity from human exposure periods, thereby reducing transmission opportunities for mosquito-borne diseases.

Our study has several limitations. Although siRNA-mediated CYC knockdown was efficient and specific, off-target effects cannot be fully excluded; future studies using genetic knockout models would strengthen causal inference. In addition, gene expression was measured at discrete time points, limiting the resolution of continuous oscillations. The neuronal basis of rhythmic behavior also remains unexplored. In addition, because sNPF was not directly perturbed, the proposed role of sNPF in shaping blood-feeding propensity remains inferred from expression patterns and known olfactory functions. Future work incorporating omics approaches, neuronal activity monitoring, and field-based experiments under natural conditions will help clarify the ecological relevance of the CYC-sNPF pathway and its utility in chrono-based vector control.

## Supporting information

S1 Table Primers utilized in the study. (DOCX)

S2 Table siRNA sequences utilized in the study. (DOCX)

S1 Fig Preliminary feeding assay data. (DOCX)

S2 Fig Individual oscillation expression data for sNPF, OR114 and OR5 under LDLD and LDDD. (DOCX)

S3 Fig Core clock genes PER, TIM, CLK expression response to extended starvation. (DOCX)

S4 Fig Day vs. Night expression contrast of core clock genes and sNPF in wild type mosquitoes. (DOCX)

S5 Fig Expression contrast of selected olfactory genes between siCYC and WT groups at daytime and night time. (DOCX)

S6 Fig Graphical summary of the study. (DOCX)
