# Supplementary material for: The circadian clock gene CYCLE as a potential target for disrupting blood-feeding behavior in the mosquito Culex pipiens
Source: PLoS Negl Trop Dis. 2026 Apr 21;20(4):e0014218. doi: 10.1371/journal.pntd.0014218 (PMC13128104; doi:10.1371/journal.pntd.0014218)
Supplement: S1 Table — (DOCX) [file pntd.0014218.s001.docx]

S1 Table. - Primers utilized in the study

| **Name** | **Sense** | **Sequence (5’-3’)** |
| --- | --- | --- |
| CLK | Forward | GGCACCGGTACAACACCTACCA |
|  | Reverse | AAGCGCATTCGGGATTTCGA |
| PER | Forward | CGCTACAAGAATGTCAACCAGCC |
|  | Reverse | ACTTGGCCGAGAGGCCCTC |
| Tim | Forward | GTCCCTTATCATTGCATTCTG |
|  | Reverse | GGTGGAATCCCAGCTTGTG |
| CQ- sNPF | Forward | TCCAACGATGACACTTTCC |
|  | Reverse | TGGGGAACATACCACCAGAT |
| CQ – OR114 | Forward | GGCCAAACCATCTCGTTGGA |
|  | Reverse | CAGCTTGTGCATGGTCAGTT |
| CQ – OR5 | Forward | AGTTAGCATTCTTAGTTCCGAT |
|  | Reverse | GAAGCACCAAGAATACTCCAC |
| RPS6 | Forward | TGATTCGCTGTTGTGTGTGTGT |
|  | Reverse | GATGTTTCGCACGCTTCG |
| CP-CYC | Forward | AGGAAATTTCACGCCGGCTA |
|  | Reverse | AACTAAACGCCAGGAGGTCA |
| CP-ctrp | Forward | GTGAGTCGTACGGAGGCTAC |
|  | Reverse | CACAACCGATACCCCACGAA |
| CP-ltrp | Forward | GGGCTTAACATCTGGTGCCT |
|  | Reverse | AACCCTCCGACGATTTTCCC |
| CP – vit1 | Forward | GGGTTGGAATAGGCGACGAA |
|  | Reverse | TGAGAACGGATTTTCGGATCTTC |
| CP - etrp | Forward | ATCGATTCTGCAAGCCGTCGT |
|  | Reverse | TCCGCCACAGATGTGATCCT |
| CQ-OR4 | Forward | ACACATGTTTACTTTCAGATCATCA |
|  | Reverse | GGGAGAGCACCCACGTTATT |
| CQ -OR56a | Forward | GCTGTACAAGAGTCAGCCGT |
|  | Reverse | CATCTCCCCAATATCGCCCC |
| CQ-OR67d | Forward | AACCATGACGATGCACGTAG |
|  | Reverse | AATGCGGTTCAGCTTGGACT |
| CQ-OR1 | Forward | AAGTCGGTACGGATCCTCAG |
|  | Reverse | TCGTTCCAATCAGCCAGGTG |
| CQ-OR2 | Forward | AAATCGAACCTCTGGCTGGG |
|  | Reverse | GCGTGCAGGTGCAGTAAAAA |
| CQ-OBP14 | Forward | CTGATGAATGCCGGTGACGA |
|  | Reverse | ACGTTTTGTTGCACTCCACG |
| CQ-OBP73a | Forward | CGGTGAAGGTGCGCATAAATC |
|  | Reverse | TAGGACCACAACCGAAACGG |
| CQ-OBP8 | Forward | GGACTCGGTCACGGAAAGTT |
|  | Reverse | TCGCATCGCTCCCATCATTT |
| CQ-OBP12 | Forward | ACGAGGCAAGATCAGCTACG |
|  | Reverse | TGAGCTACATCCTTGCAGGC |
| CQ-OBP6 | Forward | AAAGGCTTGTGCGCCAAAAT |
|  | Reverse | CGTTCCTGCCATCTGTGCTA |
