# Supplementary material for: The circadian clock gene CYCLE as a potential target for disrupting blood-feeding behavior in the mosquito Culex pipiens
Source: PLoS Negl Trop Dis. 2026 Apr 21;20(4):e0014218. doi: 10.1371/journal.pntd.0014218 (PMC13128104; doi:10.1371/journal.pntd.0014218)
Supplement: S2 Table — (DOCX) [file pntd.0014218.s002.docx]

S2 Table. siRNA sequences utilized in the study

| **Seq Id** | **Name** | **Sense** | **Anti sense** |
| --- | --- | --- | --- |
| 1 | LOC120418047-Culex pipiens pallens-775 | GGGAGAUCAGCAACCUUAUTT | AUAAGGUUGCUGAUCUCCCTT |
| 2 | LOC120418047-Culex pipiens pallens-331 | GGAUCUGCCGUACAAUAAUTT | AUUAUUGUACGGCAGAUCCTT |

The highlighted sequence showed 83% efficiency in knocking down the CYC expression and was utilized for further experiments.
