# Supplementary material for: The circadian clock gene CYCLE as a potential target for disrupting blood-feeding behavior in the mosquito Culex pipiens
Source: PLoS Negl Trop Dis. 2026 Apr 21;20(4):e0014218. doi: 10.1371/journal.pntd.0014218 (PMC13128104; doi:10.1371/journal.pntd.0014218)
Supplement: S1 Fig — (DOCX) [file pntd.0014218.s003.docx]

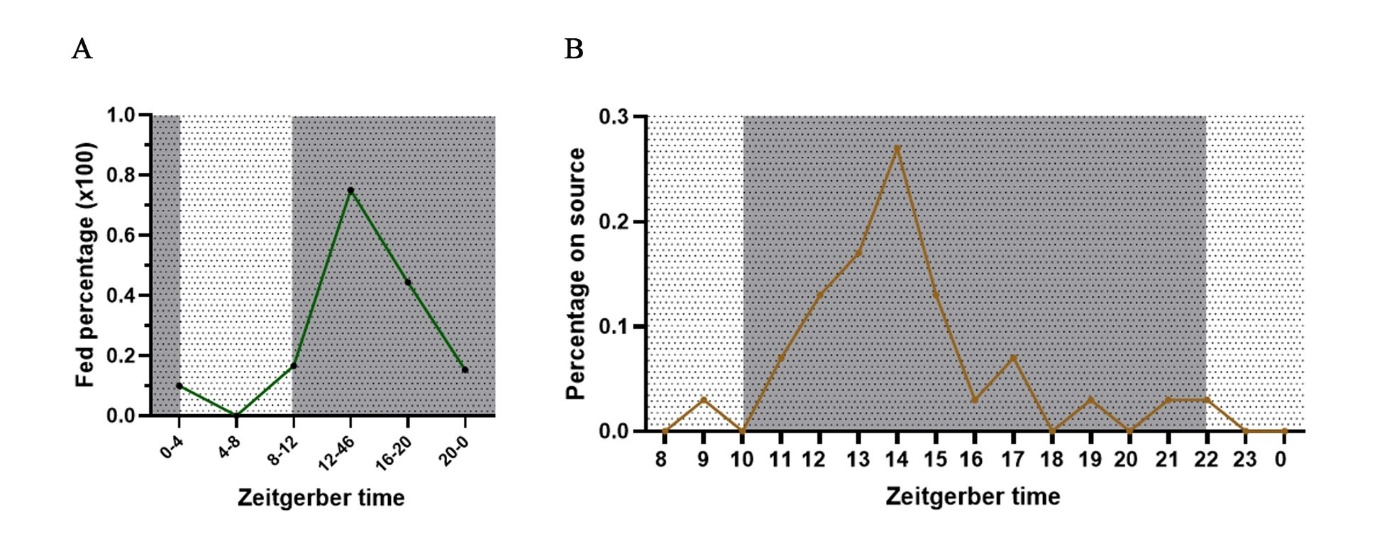


**S1 Fig**. **A** - Preliminary feeding assay data. Each reading was taken between time windows by random sampling (n=10). **B** - Percentage of female mosquitoes landed on the blood source at the time of observation. The light period was omitted due to the known inactivity of *Culex pipiens* during daytime. Background color indicates the light conditions provided: White- Lights on period, Grey - Lights off period. The experiment was purely based on observations and performed without replication, and used to interpret with known other work done on mosquitoes.
