# Supplementary material for: The circadian clock gene CYCLE as a potential target for disrupting blood-feeding behavior in the mosquito Culex pipiens
Source: PLoS Negl Trop Dis. 2026 Apr 21;20(4):e0014218. doi: 10.1371/journal.pntd.0014218 (PMC13128104; doi:10.1371/journal.pntd.0014218)
Supplement: S2 Fig — (DOCX) [file pntd.0014218.s004.docx]

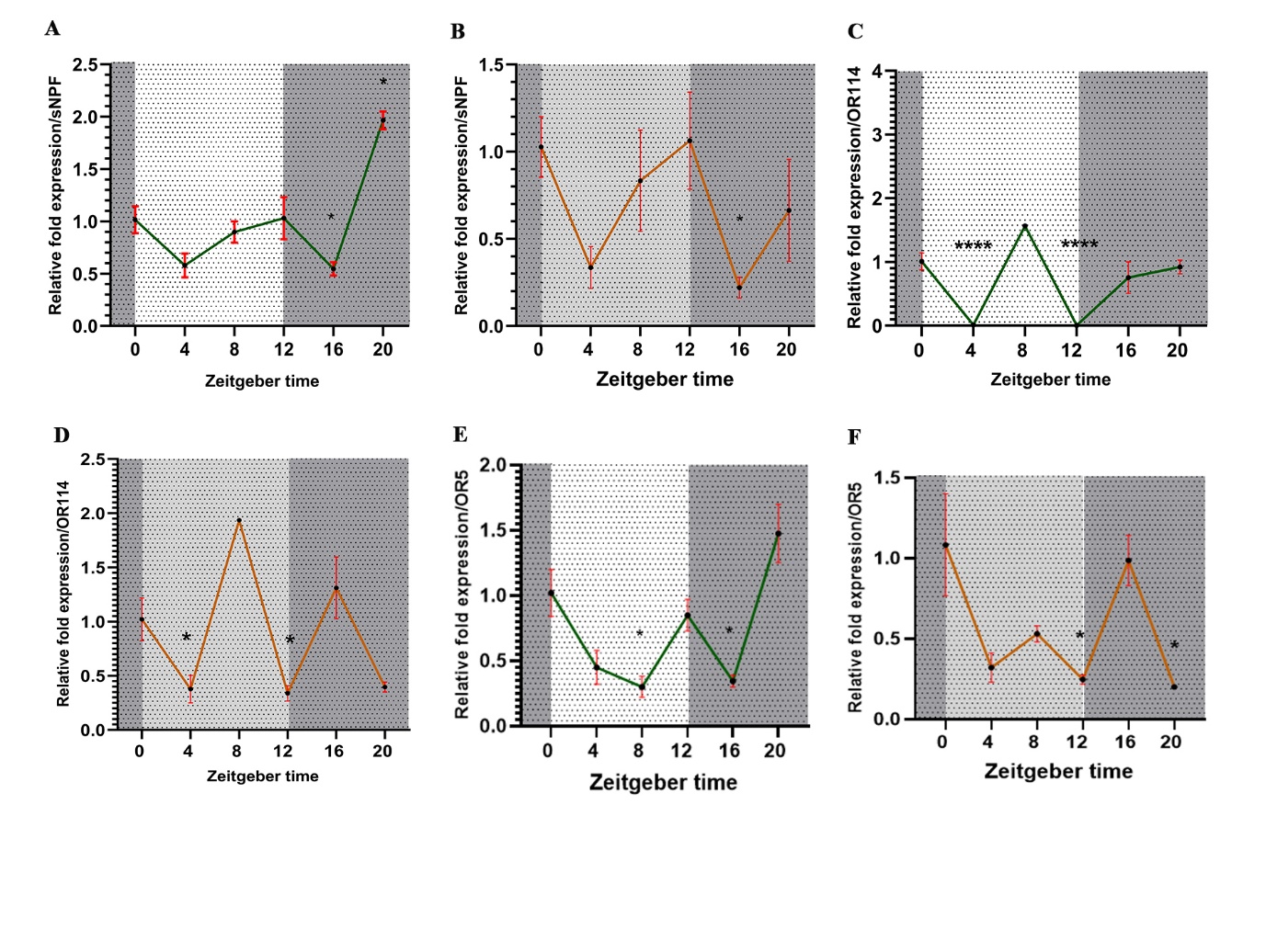


**S2 Fig. -** Individual oscillation expression data for sNPF, OR114 and OR5 under LDLD and LDDD**. A** - sNPF expression profile under LDLD conditions. **B** - sNPF expression profile under LDDD conditions. **C** - OR114 expression profile under LDLD conditions. **D** - OR114 expression profile under LDDD conditions. **E** - OR5 expression profile under LDLD conditions. **F** - OR5 expression profile under LDDD conditions. ANOVA single-factor followed by post hoc Dunnett’s test, **P*<0.05, *****P*<0.0001. Colors indicate the light conditions: White - Lights on, Dark grey – Lights off, Light grey - Lights off period under LDDD. All data are represented as Mean±SEM.
