# Supplementary material for: The circadian clock gene CYCLE as a potential target for disrupting blood-feeding behavior in the mosquito Culex pipiens
Source: PLoS Negl Trop Dis. 2026 Apr 21;20(4):e0014218. doi: 10.1371/journal.pntd.0014218 (PMC13128104; doi:10.1371/journal.pntd.0014218)
Supplement: S3 Fig — (DOCX) [file pntd.0014218.s005.docx]

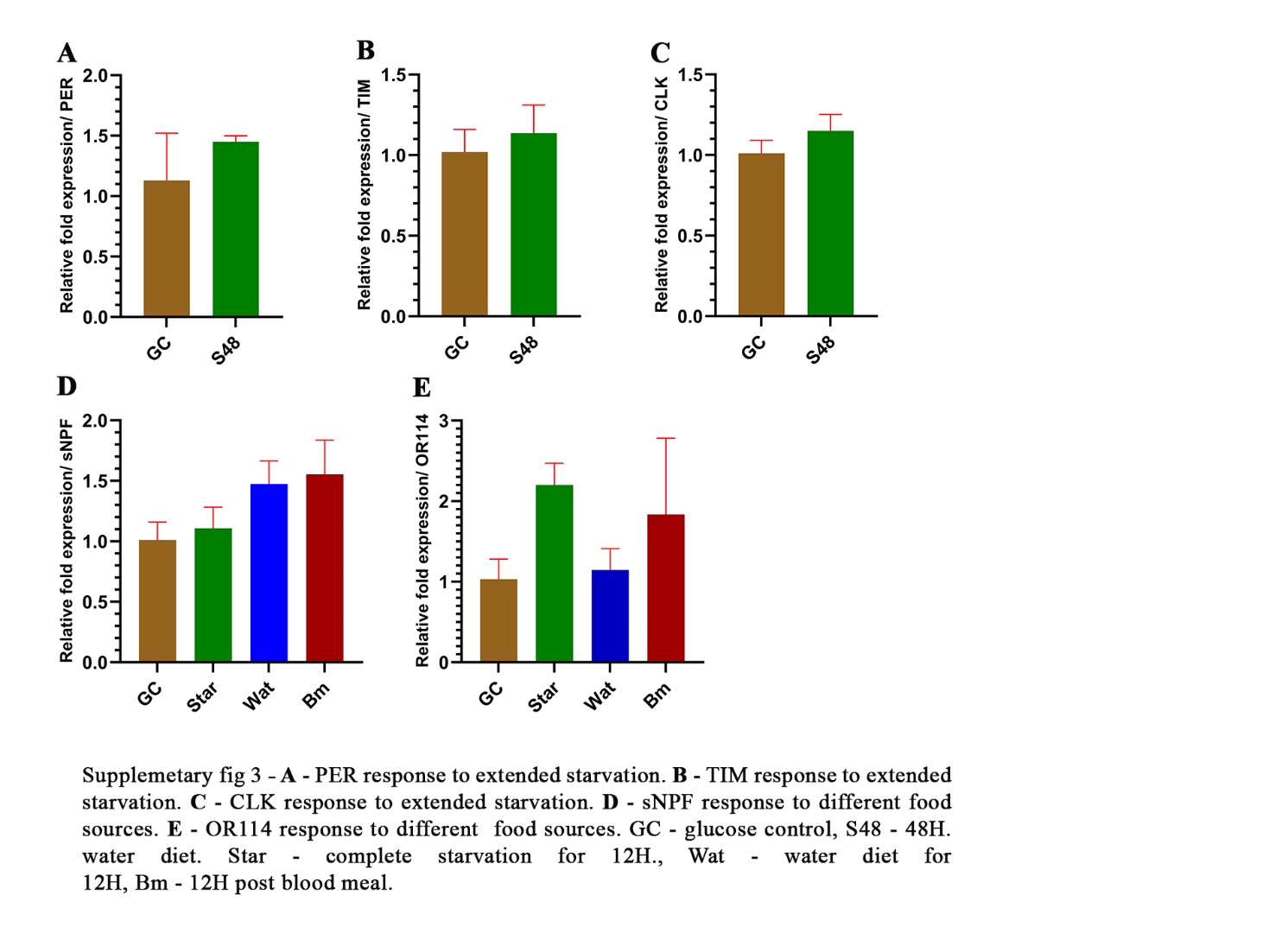
**S3 Fig.** - Core clock genes PER, TIM, CLK expression response to extended starvation**. A** - PER response to extended starvation. **B** - TIM response to extended starvation. **C** - CLK response to extended starvation. GC - Glucose control, S48 – 48hrs. water diet. Star - Complete starvation for 12 hours, Wat - water diet for 12 hours. Bm – 12 hours post blood meal. Statistical analysis performed using Student’s *t*-test. All data are represented as Mean±SEM.
