# Supplementary material for: The circadian clock gene CYCLE as a potential target for disrupting blood-feeding behavior in the mosquito Culex pipiens
Source: PLoS Negl Trop Dis. 2026 Apr 21;20(4):e0014218. doi: 10.1371/journal.pntd.0014218 (PMC13128104; doi:10.1371/journal.pntd.0014218)
Supplement: S4 Fig — (DOCX) [file pntd.0014218.s006.docx]

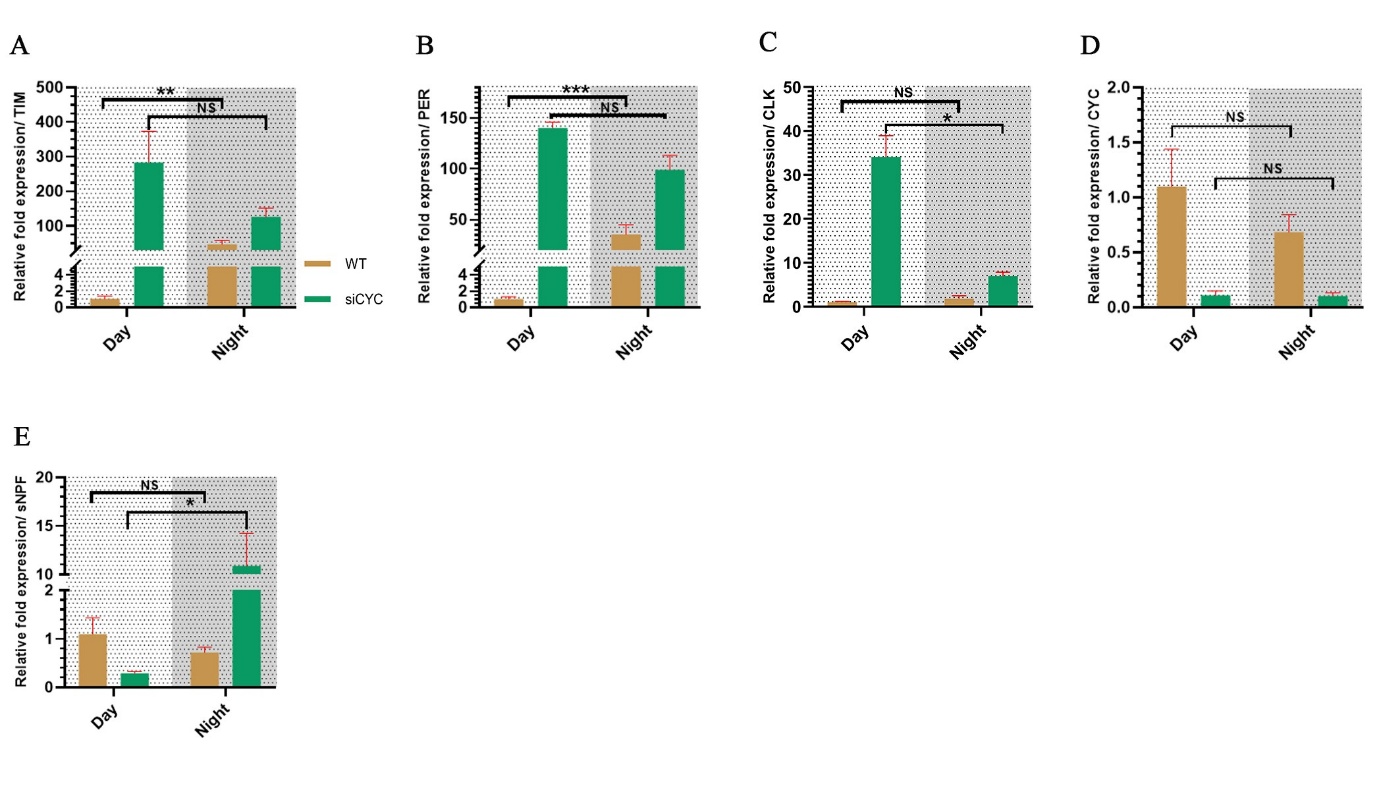


**S4 Fig.** - Day vs. Night expression contrast of core clock genes and sNPF in wild type mosquitoes. A- TIM day vs night expression contrast in WT and siCYC. B- PER day vs night expression contrast in WT and siCYC. C - CLK day vs night expression contrast in WT and siCYC. D- CYC day vs night expression contrast in WT and siCYC. E - sNPF day vs night expression contrast in WT and siCYC. Statistical analysis by ANOVA single factor followed by Tukey’s HSD. **P*<0.05, ***P*<0.01, ****P*<0.001. All data are represented as Mean±SEM.
