# Supplementary material for: The circadian clock gene CYCLE as a potential target for disrupting blood-feeding behavior in the mosquito Culex pipiens
Source: PLoS Negl Trop Dis. 2026 Apr 21;20(4):e0014218. doi: 10.1371/journal.pntd.0014218 (PMC13128104; doi:10.1371/journal.pntd.0014218)
Supplement: S5 Fig — (DOCX) [file pntd.0014218.s007.docx]

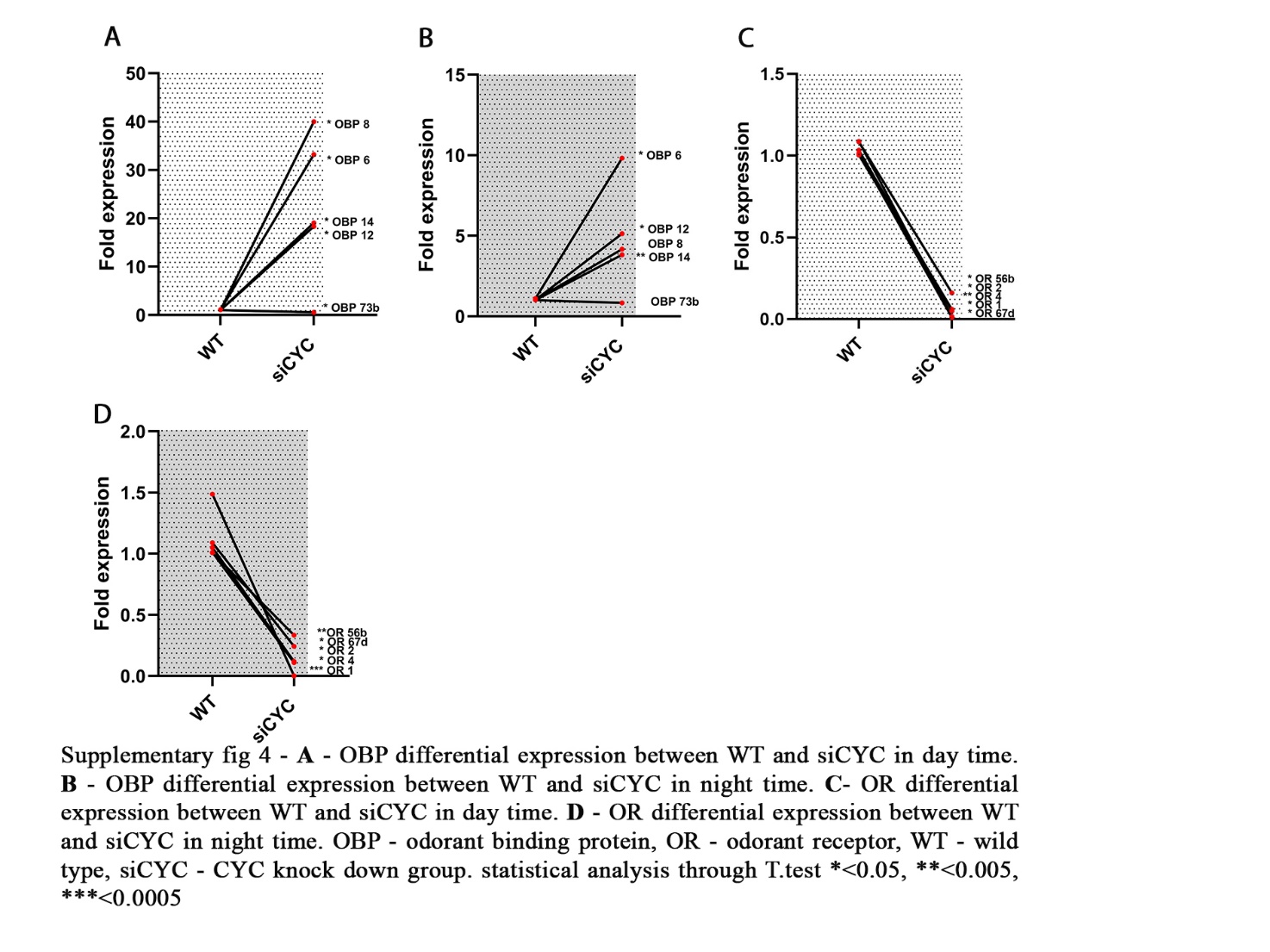
**S5 Fig.** - Expression contrast of selected olfactory genes between siCYC and WT groups at daytime and night time. **A** - OBP differential expression between WT and siCYC in daytime. **B** - OBP differential expression between WT and siCYC at nighttime. **C**- OR differential expression between WT and siCYC in daytime. **D** - OR differential expression between WT and siCYC at nighttime. OBP - Odorant binding protein, OR - Odorant receptor, WT – Wild type, siCYC - CYC knockdown group. Student’s *t*-test **P*< 0.05, ***P*< 0.01, ****P*< 0.001. All data are represented as Mean.
