# Supplementary material for: The circadian clock gene CYCLE as a potential target for disrupting blood-feeding behavior in the mosquito Culex pipiens
Source: PLoS Negl Trop Dis. 2026 Apr 21;20(4):e0014218. doi: 10.1371/journal.pntd.0014218 (PMC13128104; doi:10.1371/journal.pntd.0014218)
Supplement: S6 Fig — (DOCX) [file pntd.0014218.s008.docx]

**
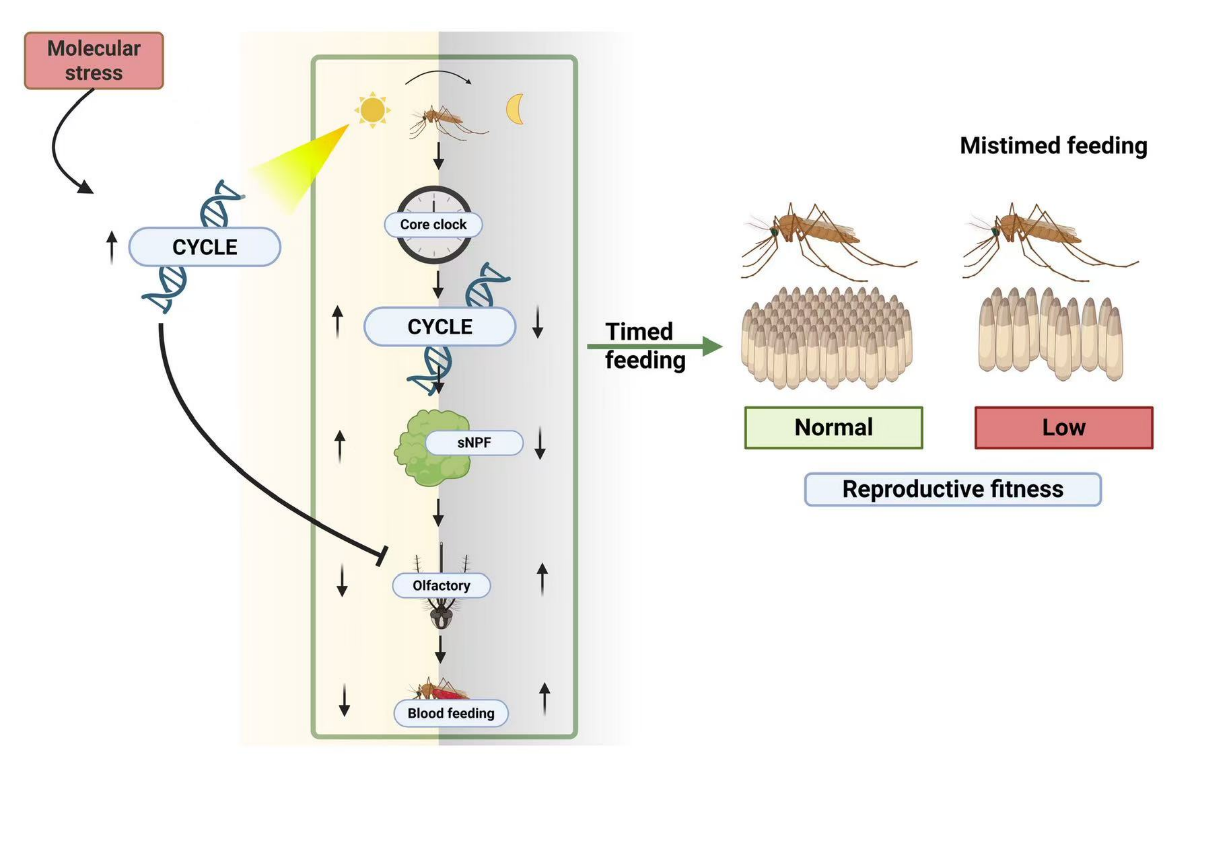
**

**S6 Fig. -** Graphical summary. Circadian clock gene CYCLE affects the blood feeding rhythm of mosquitoes by regulating the feeding rates and keep the feeding rates high at preferred time window to maintain optimal reproduction health while acting as a sensor for cellular stress to regulate energy demanding systems.
